# Facile Diastereoselective Synthesis of Dihydroxyadipic Acid and Dihydroxyadipic Dilactone by Catalytic Reduction of Biosourced 3‑Hydroxy-2-Pyrone-6-Carboxylic Acid

**DOI:** 10.1021/acsomega.5c02695

**Published:** 2025-07-07

**Authors:** Gabriella Leonardi, Aurora Bertuzzi, Ada Truscello, Cristian Gambarotti, Roberto Sebastiano

**Affiliations:** Department of Chemistry, 18981 Materials and Chemical Engineering “Giulio Natta”, Politecnico di Milano, piazza Leonardo da Vinci 32, Milano I-20133, Italy

## Abstract

3-Hydroxy-2-pyrone-6-carboxylic acid is a versatile chemical easily prepared from aldaric acids, a family of compounds obtained from renewable resources. Herein, an efficient synthesis of dihydroxyadipic acid by catalytic hydrogenation of this pyrone at room temperature and atmospheric pressure is reported. The reaction performed in organic solvents showed high diastereoselectivity, resulting in the possibility to obtain the racemic mixture of dihydroxyadipic acid (R,R) and (S,S) that was further converted to the dilactone 2,5-dioxabicyclo[2.2.2]­octane-3,6-dione by a double intramolecular condensation reaction. This compound is a promising comonomer and/or chain-extender in the synthesis of polymers. As an example, a preliminary test of the polymerization reaction of the dilactone in the presence of a diamine is reported.

## Graphical abstract

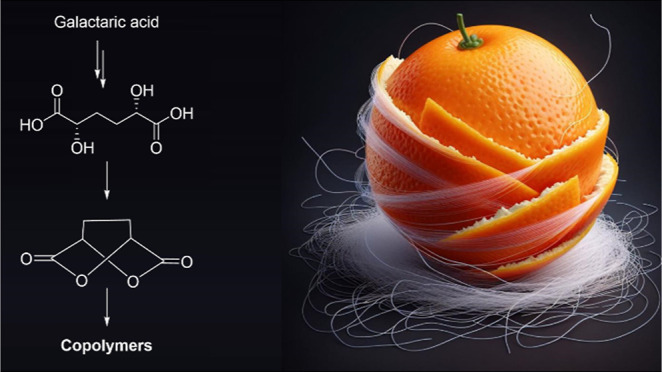

## Introduction

The transition from fossil feedstock to renewable or waste sources in the preparation of organic compounds represents one of the most important goals in the field of sustainability. In particular, the synthesis of useful platform chemicals from agriculture waste biomass is one of the hot topics in scientific community.^1–5^

In the last years, several protocols have been developed for the preparation of adipic acid (AA), an important intermediate used in the production of nylons, from renewable resources using ecofriendly processes.^6–15^ Recently, aldaric acids, and their derivatives, have been studied as precursor of biobased AA.^16–19^ 2,5-Dihydroxyadipic acid (DHAA) was found to be an intermediate for the synthesis of tetrahydrofuran-2,5-dicarboxylic acid (THFDCA, Figure 1a)^20,21^ that in turn is a precursor of AA.^10,22^ Also, in a recent patent, DHAA itself is claimed to be a precursor of AA.^23^

**Figure 1:**
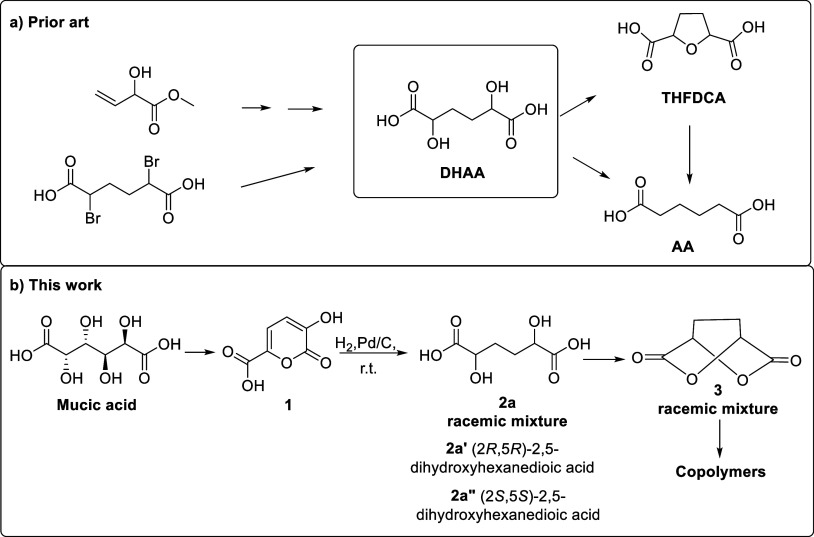
Synthesis and use of DHAA: (a) prior art and (b) this work.

The use of DHAA is not limited to AA preparation as itself can be used as a monomer in the synthesis of polymers.^24^

The preparation of DHAA from adipic acid through the intermediate dibromo derivative was reported a century ago (Figure 1a).^25^ The meso DHAA was also obtained, together with other coproducts, from gluconic acid in the presence of barium hydroxide at 140 °C.^26^ Recently, DHAA has been prepared from sugar-derived methyl vinyl glycolate (Figure 1a),^24^ by hydrogenation of aldaric acids over Pd/TiO_2_ performed at 2 MPa and 150 °C^23^ and from 1,2,5,6-hexanetetrol in the presence of a platinum–bismuth.^20^

In recent years, our research group has been involved in the valorization of aldaric acids since these compounds are interesting polyfunctional molecules derived from polysaccharides present in biomass. In this context, we developed an efficient and ecofriendly synthesis of the pseudo aromatic 3-hydroxy-2-pyrone-6-carboxylic acid (3-hydroxy-2-oxo-2*H*-pyran-6-carboxylic acid, **1**) from galactaric acid (mucic acid) (Figure 1b).^27^ This compound has proven to be a versatile precursor in the synthesis of various aromatic compounds.^28,29^

In this paper, the catalytic reduction under mild conditions of biosourced 3-hydroxy-pyrone **1** is reported (Figure 1b). The reaction performed in water led to a diastereoisomeric mixture of DHAA, whereas, in organic solvents, an almost complete diastereoselectivity was observed, obtaining, by hydrolysis, the racemic mixture (2*R*,5*R* and 2*S*,5*S*) of 2,5-dihydroxyhexanedioic acid (**2a**) (Figure 1b). Having raceme DHAA **2a** the suitable configuration to allow the ring closure, it was possible to obtain the corresponding dilactone 2,5-dioxabicyclo[2.2.2]­octane-3,6-dione (**3**) (Figure 1b).^25,30^ Dilactone **3**, already used as a comonomer in the polymerization with lactide,^31^ could be also used as a comonomer in the polymerization with diols or diamine to obtain biosourced polyhydroxyesters or polyhydroxyamides^32^ and as a cross-linking agent. As an example, a preliminary test of polymerization of **3** with hexamethylenediamine is reported.

## Results and Discussion

With the aim to perform the catalytic hydrogenation of hydroxypyrone **1** under mild and green conditions, we verified the possibility of carrying out the reaction in water at room temperature in the presence of hydrogen at atmospheric pressure and Pd/C as a catalyst. DHAA **2** was obtained in 90% yields within 18 h (Scheme 1). The structure was confirmed by comparison of ^1^H NMR spectrum with the data reported in literature.^24^

**Scheme 1:**
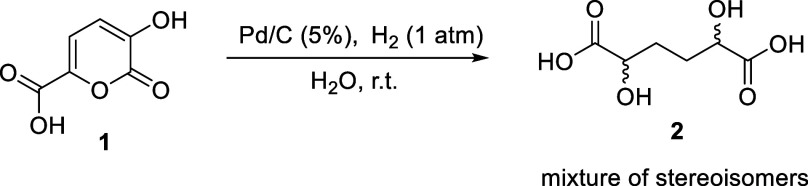
Hydrogenation of **1** in Water

The formation of DHAA **2** can proceed by following the steps reported in Schemes 2 and 3. The first addition of hydrogen on pyrone **1** can occur from the “bottom” (Scheme 2, a) on the γ-δ double bond giving **4a′** and/or on α–β double bond giving **4b′** and respectively **4a’’** and/or **4b’’** from the “top” (Scheme 2b).

**Scheme 2:**
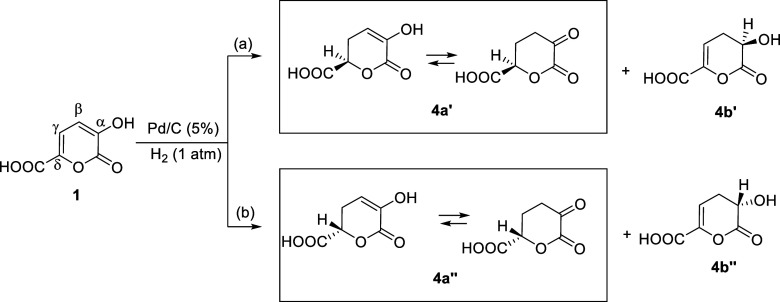
First Addition of Hydrogen on Pyrone **1** from the “Bottom” (a) and from the “Top” (b)

**Scheme 3:**
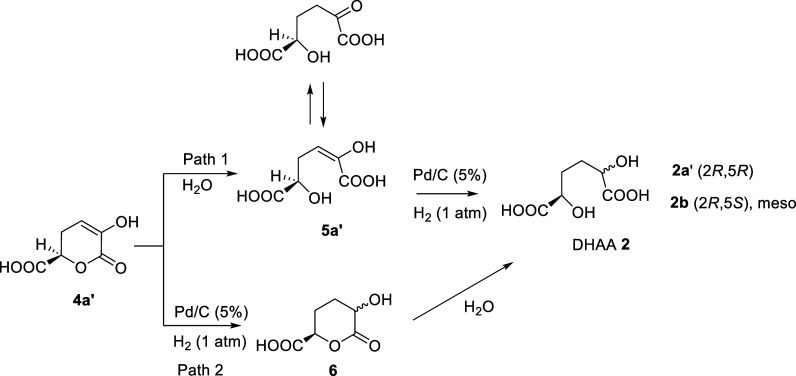
Possible Evolution of **4a′** to Dihydroxyadipic Acid **2** (See Scheme S1 for **4a’’**)

As far as we know, in literature, it is generally reported that the first addition of hydrogen on pyrones occurs on the γ-δ double bond,^33–35^ but in those cases pyrone atoms in γ and δ are not substituted with groups that could give resonance effect with the double bond. For this reason, in our case, we cannot exclude the attack of hydrogen on the α–β double bond because of the presence of a carboxylic group.

Performing the reaction in water, the further hydrogenation of monounsaturated lactone **4a′** can proceed through two different pathways (Scheme 3). Following path 1, **4a′** initially undergoes hydrolysis to give the corresponding diacid **5a′**, followed by hydrogenation obtaining the target DHAA as mixtures of diastereoisomers. In path 2, the second hydrogenation occurs before the hydrolysis of **4a′** forming the two diastereoisomer **6**. The enantiomer **4a’’** would give the formation of the corresponding **2a”** (2*S*,5*S*) and meso **2b** (2*S*,5*R*) (Scheme S1). Similarly, monolactones **4b′** and **4b’’** can follow the same paths to give **2** (Scheme S2).

In principle, based only on the statistical distribution, the mixture should be composed by 50% of the raceme DHAA **2a** (**2a’** = 25%, **2a”** = 25%) and 50% of meso DHAA **2b**.

In literature, it is reported that hydrogenation of similar pyrones can be diastereoselective depending on the substrate and the reaction conditions.^36,37^ Therefore, an investigation of the stereoselectivity of the reaction was performed.

The quantitative ^13^C NMR analysis of the reaction mixture obtained by hydrogenation in water (Figure 2) showed a 1:2 ratio of the two DHAA diastereoisomers.

**Figure 2:**
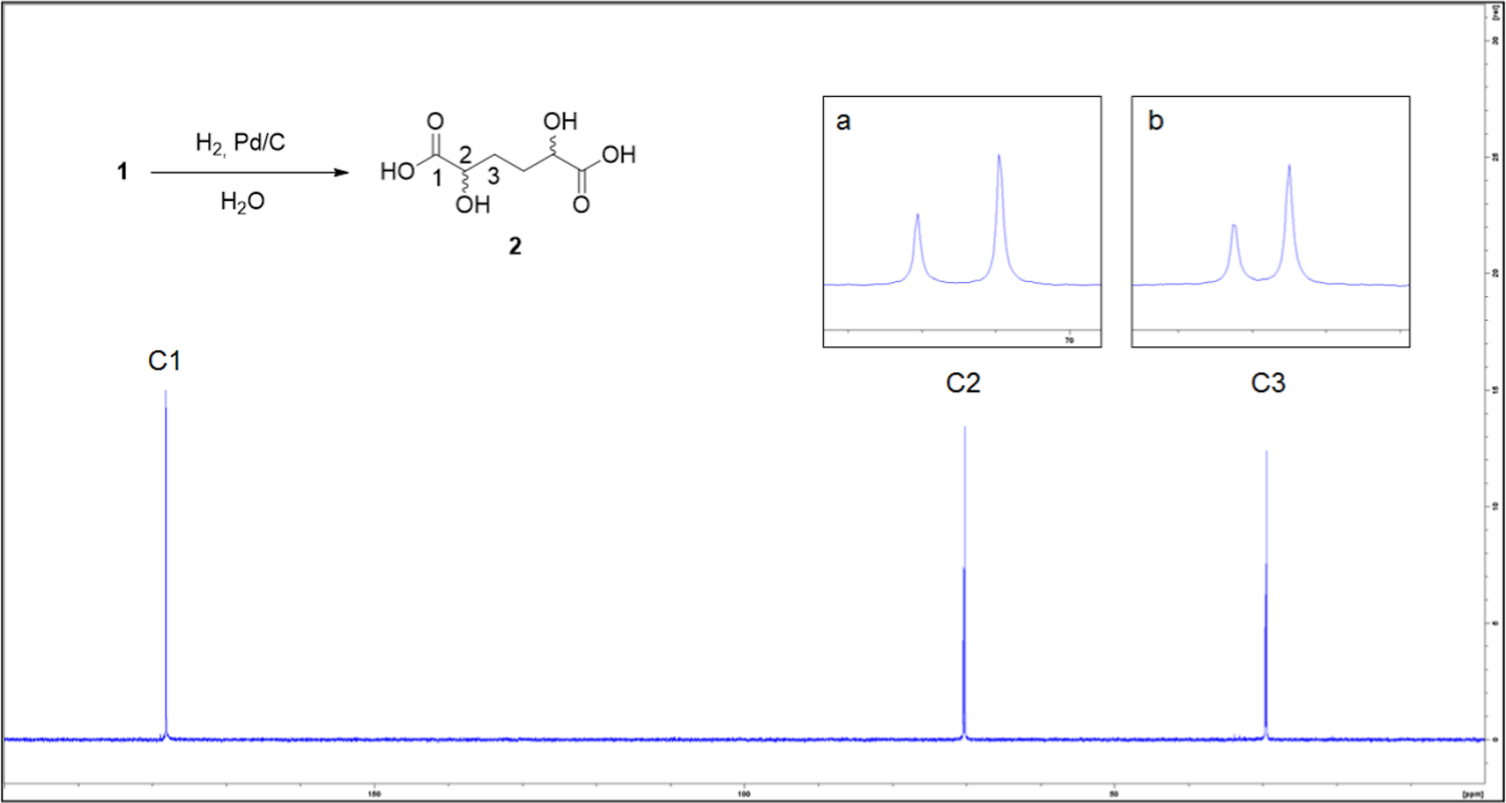
^13^C NMR (D_2_O) spectrum of 2 obtained by hydrogenation of 1. (a) Expansion of the region of C2; (b) expansion of the region of C3.

The identification the two diastereoisomers of DHAA **2** was performed by preparing the corresponding dilactone **3** by the thermal intramolecular condensation reaction (Scheme 4).^25^ Dilactone **3** can be obtained only by the double lactonization of diastereoisomer **2a** (racemic mixture of **2a′** and **2a”**), via the formation of the intermediate monolactone **6a** (racemic mixture of **6a′** and **6a”**), as the OH and COOH substituents are in *syn* configuration. Conversely, the meso form **2b** can afford only the monolactone **6b** (racemic mixture of **6b′** and **6b”**) (Scheme 4).

**Scheme 4:**
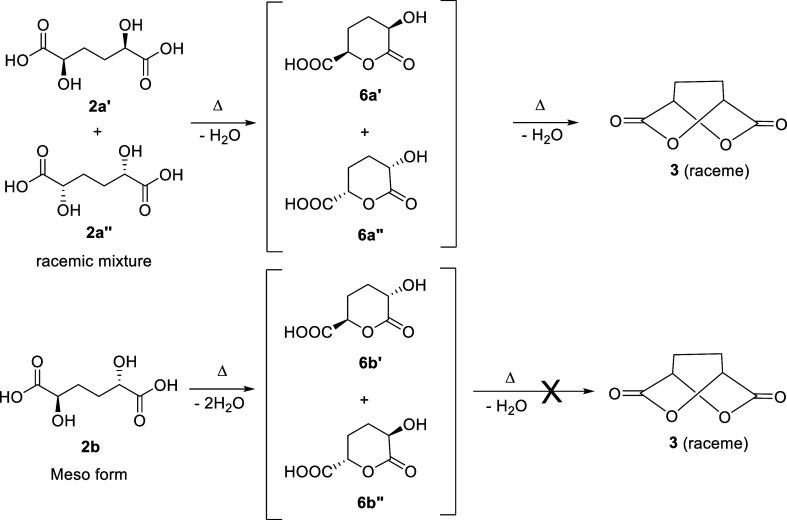
Lactonization of DHAA Diastereoisomers

Differently from that reported in the literature^25^ where only a thermal treatment was used, we perform the lactonization by heating in a tinyclave the DHAA **2** dissolved in acetic acid. Dilactone **3** was then isolated by sublimation, analyzed by NMR, and identified by comparison with the data reported in literature.^30^

Dilactone **3** was then hydrolyzed in aqueous hydrochloric acid to form the corresponding DHAA **2a**. By the ^13^C NMR spectrum of **2a** (Figure 3b), it was possible to identify and assign the signals of the carbon bearing the hydroxyl group (C2) and the carbon of the methylene group (C3). Therefore, the signals present in the spectrum of the mixture of the two diastereoisomers **2** (Figures 2 and 3a), obtained by hydrogenation of **1**, can be correctly assigned.

**Figure 3:**
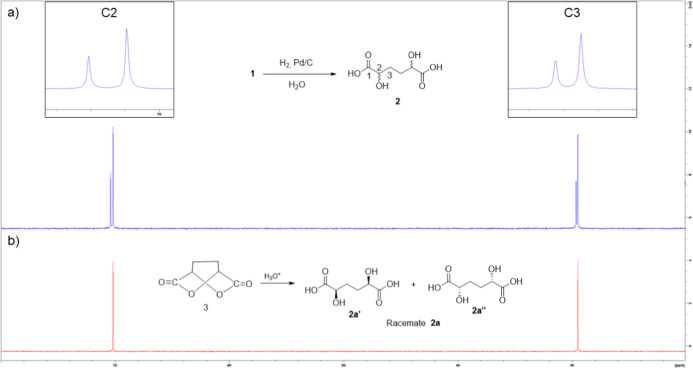
^13^C NMR (D_2_O) spectra of (a) hydrogenation of pyrone **1** in water; (b) hydrolysis of dilactone **3**.

This result indicates that the major product obtained in the catalytic reduction in water of pyrone **1** is represented by the racemic mixture of two enantiomers **2a′** and **2a’’**.

It is known that the solvent can affect the selectivity of the hydrogenation of pyrones.^36,37^ In particular, as reported in Scheme 3, water can promote hydrolysis as well as the desorption from the catalyst. For these reasons, we investigated the reaction under the same conditions (room temperature and hydrogen at ambient pressure) in nonhydrolytic solvents such as acetic acid, THF, and ethanol.

Reactions were carried out up to no consumption of hydrogen, and the crude mixtures were analyzed by ^1^H NMR spectroscopy after removal of the solvent. Interestingly, the ^1^H NMR spectra of the reactions performed in THF or acetic acid showed the presence of a monolactone together with dilactone **3** and DHAA **2** (see Supporting Information, Figures S9 and 10). It has to be underlined that the ratios of these products, especially for experiments in acetic acid, depend upon the work up conditions as lactones are sensitive to temperature and moisture; for the same reason, all attempts in isolating monolactone failed. Thus, in an experiment performed in acetic acid, in which the catalyst was previously treated with acetic anhydride in order to remove moisture, two samples were withdrawn and the solvent was removed, respectively, at 70 °C under reduced pressure and at room temperature under a stream of nitrogen. The ^1^H NMR analyses (see Supporting Information, Figure S11) showed the monolactone as the major product in the sample treated at room temperature, whereas the dilactone was the major product present in the sample treated at 70 °C. Therefore, as the monolactone converted into **3** on heating, its structure was assigned to racemic monolactone **6a**.

The reaction was performed in ethanol (Figure S12), resulted in an almost total conversion of **1** into the monoester **7** (Figure 4), and this latter was isolated and identified by NMR and MS spectroscopies.

**Figure 4:**
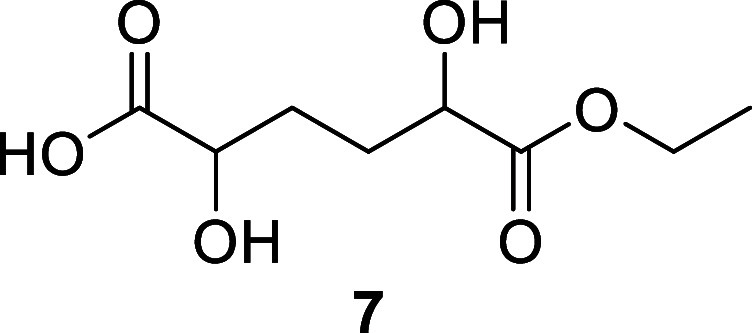
Structure of monoester **7**.

Being the DHAA **2** the target product, the mixtures of hydrogenations in THF, acetic acid, and ethanol, after the removal of catalyst and solvents, were submitted to hydrolysis. In Table 1, the overall isolated yields and the diastereoisomeric ratio **2** *a* **/2b**, determined by quantitative ^13^C NMR analysis, are reported.

**Table 1:**
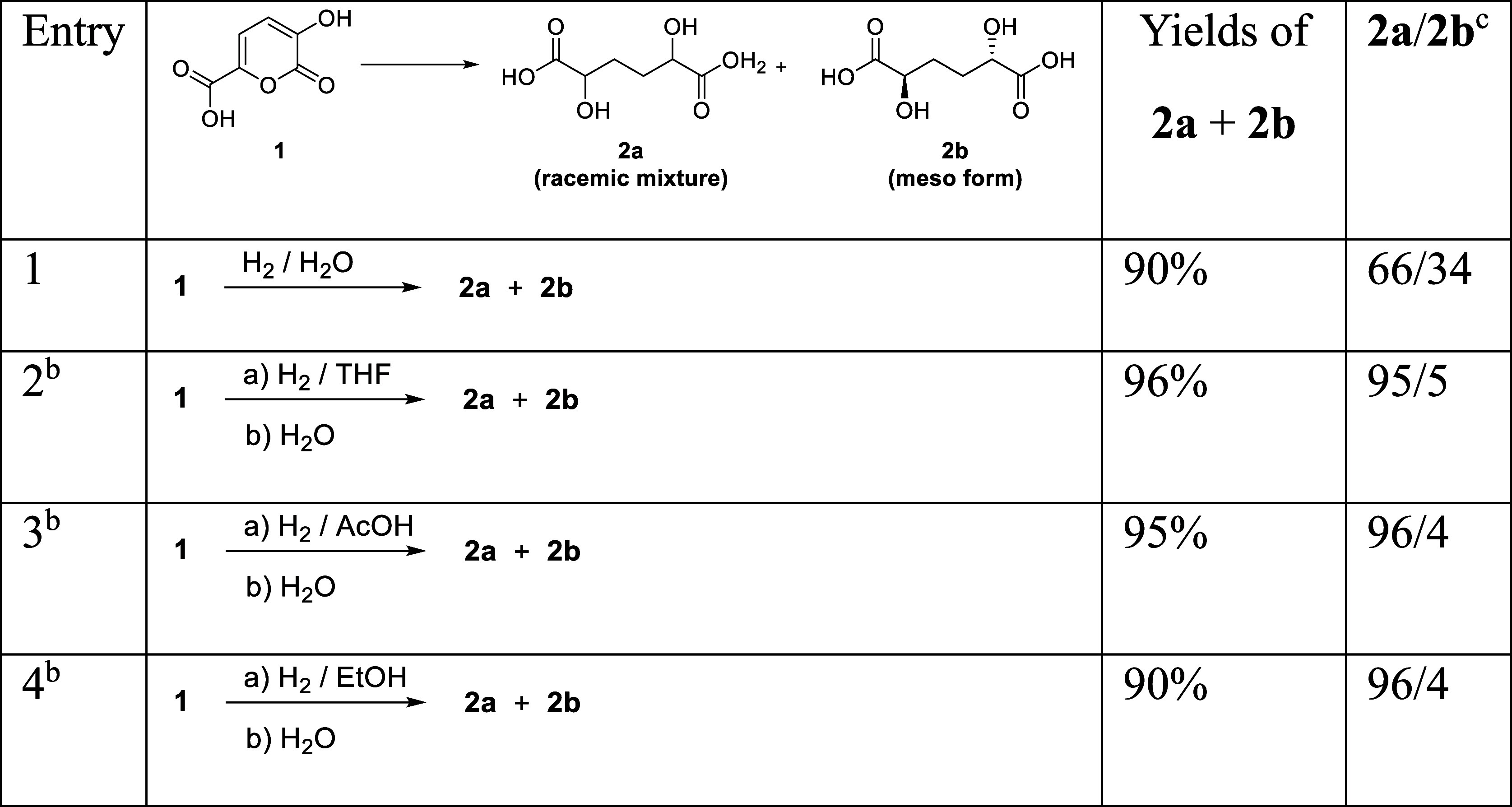
Hydrogenation of Pyrone **1** ^a^: Isolated Yields of DHAA **2** (**2a + 2b**) and Diastereoisomeric Ratios **2** *a* **/2b**

a Hydrogenation performed at 1 atm, room temperature in the presence of 5% Pd/C.

b Hydrolyses were performed under acidic conditions. Details are given in the experimental part.

c Ratios determined by quantitative ^13^C NMR analysis; ratios were calculated using the average value of the two corresponding peaks around 70 and 30 ppm (Figure S13).

Reactions performed in organic solvents (entries 2–4) showed, after hydrolysis, substantially the same yields and the same very good diastereoselectivity toward the racemic dihydroxyadipic acid **2a**. The observed diastereoselectivity is comparable to that reported in literature for similar pyrones^36,37^ except in the case of alcoholic solvent, where no selectivity is reported.^37^

With the aim to monitor the hydrogenation during the time, the reaction in THF was also performed using a continuous H-Cube microhydrogenator apparatus, operating at 10 bar and 30 °C in the presence of 10% Pd/C. In this case, it was possible to analyze by ^1^H NMR spectroscopy the reaction mixture (see Supporting Information, Figure S14) just after removing the solvent at reduced pressure. The percentage of unconverted pyrone **1** and the analytical yields of the products were calculated by ^1^H NMR in the presence of an internal standard and are reported in Table S1 (see the Supporting Information). The corresponding data plots of **1** and **6a** (racemic mixture of **6a′** and **6a’’**) are drawn in Figure 5. Monolactone **6a** is substantially the only product present in the reaction mixture. Only small amounts of DHAA **2** (1–2%) and dilactone **3** (1%) were identified in the ^1^H NMR spectra of the mixture, and their formation was probably due to the work-up procedure.

**Figure 5:**
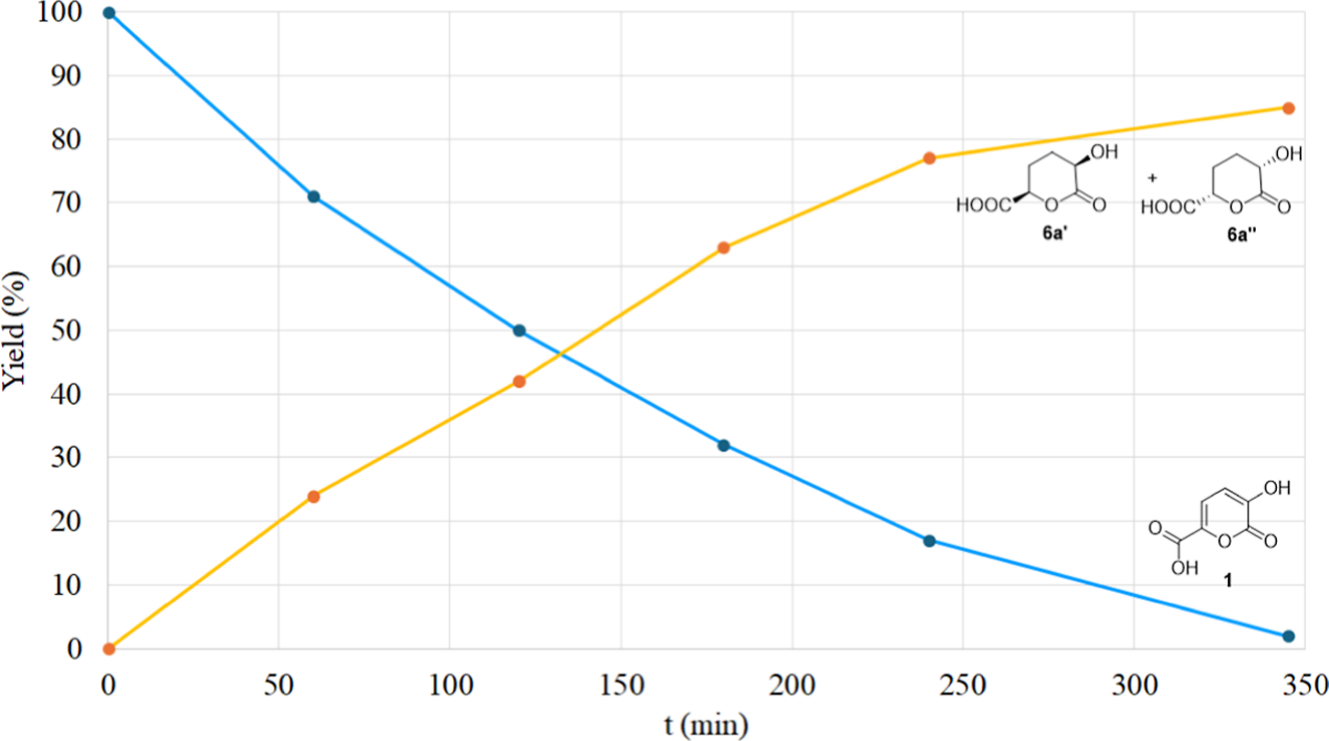
Hydrogenation of **1** performed in the H-Cube apparatus (H_2_ 10 bar, 10% Pd/C, THF, 30 °C).

At low conversion, only very small signals, probably due to traces of dihydropyrone intermediates, were detected in the spectrum. Therefore, hydrogenation of these intermediates seems to be faster than that of the pyrone itself. As **6a** was by far the major component after 345 min, the ^1^H NMR analysis allowed the assignment of the corresponding signals.

The diastereoselectivity **2a**/**2b** observed (Table 1) can be justified considering the reaction mechanisms reported in Scheme 5. In order to simplify the discussion, only the enantiomers *R* (**4a′**) and *R,R* (**6a′**) are considered; their corresponding enantiomers **4a’’** and **6a’’** lead to the corresponding products (see the analogous Scheme S3 in the Supporting Information).

**Scheme 5:**
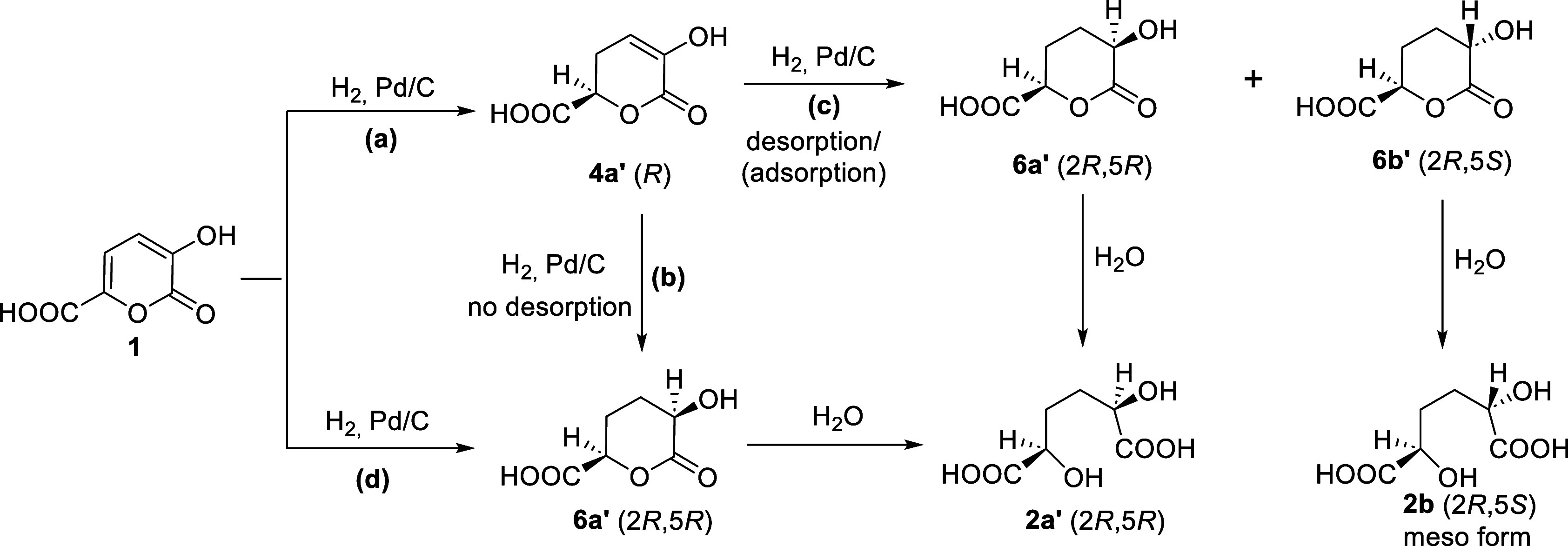
Catalytic Hydrogenation of **1**: General Scheme

In the mechanism proposed, the adsorption/desorption processes of intermediate **4a′** from the catalyst surface are the major responsible of the diastereoselectivity. These processes depend on the nature of the solvent.

Regardless of the solvent used, the hydrogenation of **1** can occur following different paths: (i) formation of intermediate **4a′** followed by hydrogenation to give **6a′** without desorption from the catalyst surface (Scheme 5a,b); (ii) formation of intermediate **4a′** and further hydrogenation to give **6a′** and **6b′** through desorption/adsorption from the catalyst surface (Scheme 5a,c); and (iii) simultaneous hydrogenation of the two double bonds of **1** to give **6a’** (Scheme 5d).

When water is used as the solvent, the hydrolysis of **4a′** to give **5a′** cannot be excluded as previously discussed (Scheme 3). Anyway **5a′** would evolve giving the same products through similar mechanisms.

Although the intermediate **4a′** has not been identified, its formation has to be considered as it allows us to justify the partial diastereoselectivity observed, in particular in the case of the reaction in water. In fact, the presence of the two diastereoisomers **2a** and **2b** can be explained only by assuming that part of **4a′** undergoes desorption from the catalyst and then adsorption on the other face of the ring. Thus, the further hydrogenation affords derivative **6b′** resulting in the observed loss of diastereoselectivity (Scheme 5c).

As the hydrogenation of **1** performed in THF and in acetic acid (Table 1, entries 2,3 and Figure 5), after hydrolysis, showed a very high diastereoselectivity in **2a**, the a, b, and d reaction pathways (Scheme 5) are the preferred ones. Conversely, when hydrogenation of **1** is carried out in water (Table 1, entry 1), low diastereoselectivity is observed (**2a**/**2b** ratio of 66:34), which indicates that the a and c reaction paths (Scheme 5) become competitive.

Similar to that discussed above, same products would be obtained also from **4b′** or **4b’’** (Scheme 2) and mechanism for **4b′** is proposed in the Supporting Information (Schemes S4).

As reported above, dilactone **3** was prepared in 12% yield starting from the mixture 66:34 of **2a**/**2b**. Therefore, an attempt to maximize the yield of **3** was performed by running the dehydration of the reaction mixture containing almost pure **2a** in a tinyclave at 140 °C using acetic acid as the solvent, obtaining **3** in 60% yield.

The dilactone **3** can represent a useful platform for the synthesis of copolymers as well as a branching agent. In this context, a very preliminary test of polymerization has been performed just to evaluate the possibility to use **3** as a comonomer. The reaction of dilactone with hexamethylenediamine **8** was performed at room temperature in DMSO-*d*_6_ (Scheme 6).

**Scheme 6:**
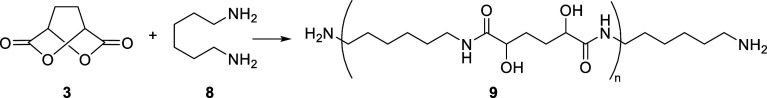
Polymerization Reaction of **3** with Hexamethylenediamine **8**

^1^H NMR analysis (Figure 6) after 24 h showed the presence of a mixture of products; the structure of the major component is compatible with the structure **9**.

**Figure 6:**
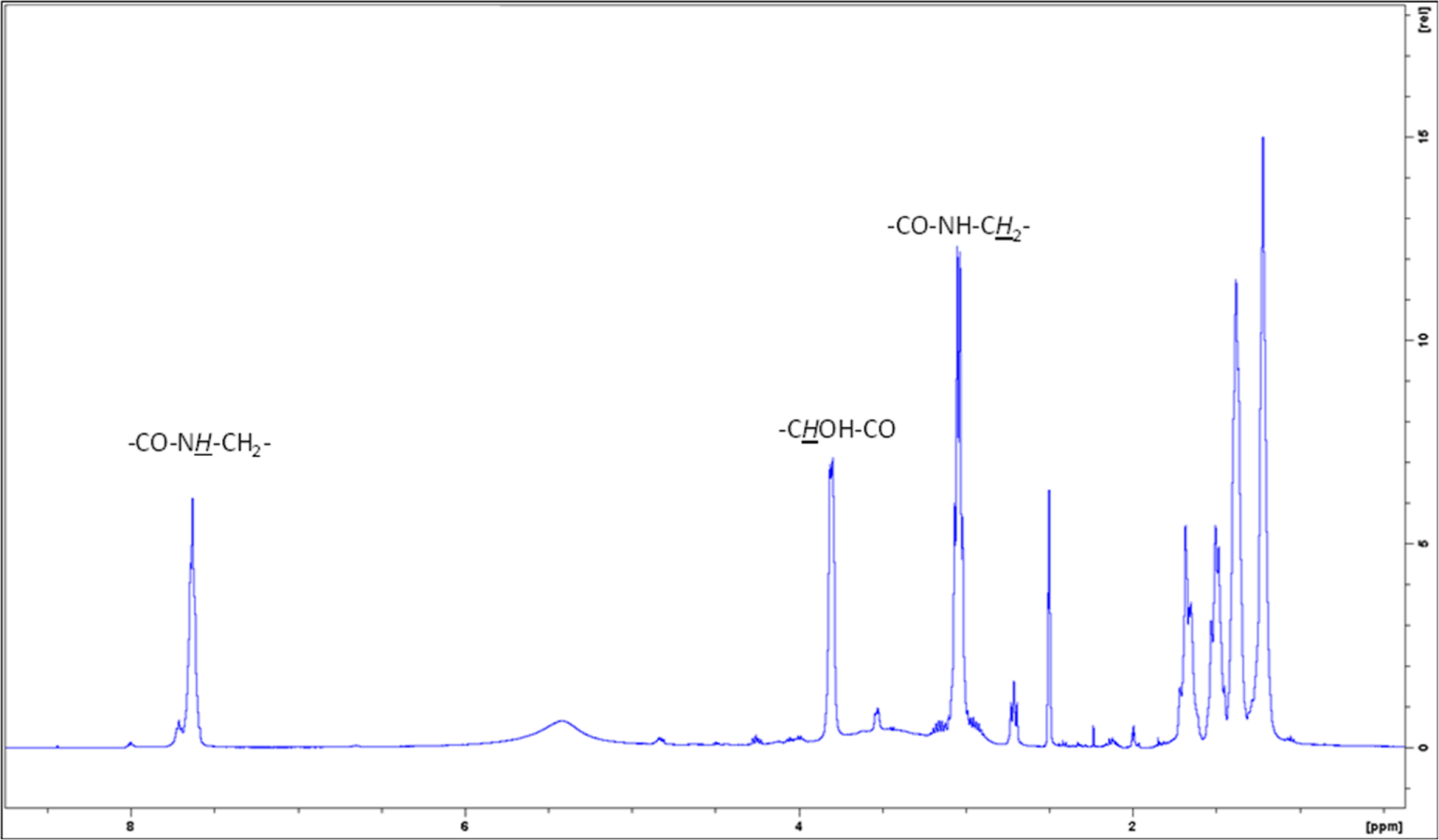
^1^H NMR spectrum (DMSO-*d*_6_) of the polymerization mixture.

In the ^1^H NMR spectrum of **9** (Figure 6), the typical NH signal of the amide appears at 7.63 ppm, whereas at 3.80 ppm, there is a signal that could be assigned to the protons bound to the hydroxyl groups in the molecule. The methylene in α to NH of the amide appears at 3.04 ppm, whereas the small signals at 2.71 ppm could be assigned to the methylene protons bound to the nitrogen atom of the ending groups. The fact that the NH_2_ function has been found at both the end groups of structure **9** is suggested by the ESI-MS spectrum of the crude of polymerization reaction. The MS spectrum of the reaction mixture (Supporting Information, Figure S16) showed that the major part of the signals is that of **9** (*n* = 1–9), whereas only two signals seem to be ascribed to the presence of **10** (*n* = 1,2) (Figure 7). Anyway, in the ^1^H NMR spectrum, the signals compatible with the presence of **10** are very low.

**Figure 7:**
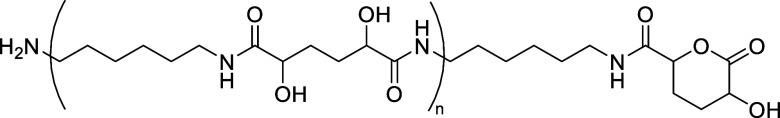
Structure of the product with lactone as the end group **10**.

The ^13^C NMR spectrum (Supporting Information, Figure S15) of the reaction mixture was recorded. The signal at 174.7 ppm confirmed the presence of the amide moiety.

In the ATR-FTIR (Supporting Information, Figure S17) of the solid precipitated from the reaction mixture, the peak at 1630 cm^–1^ can be ascribed to the amide C=O stretching, whereas the peak at 1534 cm^–1^ is ascribed to the bending of the amide NH bond.

## Conclusions

The very high diastereoselective synthesis of dihydroxyadipic acid starting from biosourced 3-hydroxy-2-pyrone-6-carboxylic acid **1** has been performed. The hydrogenation of **1** has been carried out in organic polar solvents in the presence of Pd/C under hydrogen at atmospheric pressure, at room temperature and without the need of asymmetric ligands, obtaining intermediate lactones that, after hydrolysis, led to the (*R*,*R*) and (*S*,*S*) racemic mixture of DHAA. The protocol showed a diastereoselectivity higher than 95%. A lower diastereoselectivity in the hydrogenation of **1** in water was found.

From the racemic mixture, it was possible to prepare the corresponding dilactone, a promising comonomer in the synthesis of polymers, and as an example, a preliminary test of the polymerization reaction in the presence of hexamethylenediamine is reported.

An attempt to discuss the very high diastereoselectivity observed is disclosed, and a mechanism which includes the possibility to have hydrogenation of the two double bonds of the pyrone without desorption from the catalyst is proposed.

## Methods

A tinyclave Büchiglass—Büchi AG (internal diameter of 2.5 and 10 cm length) was used for preparation of dilactone **3**. A H-Cube Miniplus ThalesNano apparatus was used for the hydrogenation in THF. A Bruker AV 400 MHz instrument was used to record ^1^H NMR (400 MHz) and ^13^C NMR (100.6 MHz) spectra (100.6 MHz). ^1^H chemical shifts (δ) are given in parts per million relative to the residual proton of the solvent. ^13^C chemical shifts (δ) are given in ppm relative to the signal of the solvent; in the case of D_2_O, to the signals of DMSO (39.39 ppm) added to the sample as an internal reference. Quantitative ^13^C NMR spectra were acquired setting D1 = 20 s. MS analyses were performed with an Esquire 3000 plus ion-trap mass spectrometer equipped with an ESI source and with an Agilent 6420 Triple Quadrupole LC/MS system 6420 equipped with an ESI source. Melting points were determined with a Büchi 535 instrument and are uncorrected. ATR-FTIR spectrum was recorded using a Thermo Scientific Nicolet iS5 FT-IR Spectrometer with Nicolet id7 ATR unit (4000–400 cm^–1^ range). The collected data are analyzed with Thermo Scientific software OMNIC. Pyrone **1** was prepared from mucic acid by the procedure reported.^27^

### Hydrogenation of **1** in Water

In a three-necked round-bottomed flask, 1.31 g of 3-hydroxy-2-pyrone-6-carboxylic acid (**1**) (purity 90%, 7.57 mmol) was dispersed in 26 mL of water. Then, 0.66 g of 5% Pd/C (50% moisture) was added to this suspension, and the resulting mixture was stirred at room temperature under the hydrogen atmosphere (ambient pressure) until no further hydrogen consumption was observed (18 h). The reaction mixture was then filtered, and the solvent was removed under reduced pressure to obtain 1.30 g (purity 94%, 90% yield) of **2** as a white solid. ^1^H NMR (DMSO-*d*_6_): δ 3.98–3.88 (m, 2H), 1.78–1.65 (m, 2H),^24^ 1.64–1.50 (m, 2H); ^1^H NMR (D_2_O, 4.79 ppm): δ 4.40–4.32 (m, 2H), 2.05–1.91 (m, 2H), 1.91–1.76 (m, 2H); ^13^C NMR (D_2_O + DMSO as reference 39.39 ppm): δ 178.1, 178.1, 70.4, 70.2, 29.7, 29.5.

### Synthesis of Dilactone **3** by Thermal Dehydration of **2**

In a 25 mL tinyclave, DHAA **2** (obtained by hydrogenation in water) (0.512 g, purity 94%, 2.71 mmol) was dissolved in 15.0 mL of acetic acid. The solution was heated at 150 °C (oil bath) under stirring for 1 h and 50 min. After this time, the mixture was cooled to room temperature, and the solvent was removed under reduced pressure, obtaining 0.489 g of a white sticky solid. A sample of the solid was analyzed by ^1^H NMR spectroscopy in the presence of terephthalic acid as an internal standard. The analysis showed the presence of **3** in a 46% yield. The solid was then loaded in a sublimation apparatus and heated at 140 °C (oil bath), recovering **3** as a white solid (0.046 g, 0.324 mmol, yield 12%). *M*_p_ 129–131 °C;^25,30^ ^1^H NMR (CDCl_3_): δ 5.01–4.99 (m, 2H), 2.44–2.33 (m, 2H), 2.25–2.13 (m, 2H);^23^ ^1^H NMR (DMSO-*d*_6_): δ 5.28–5.24 (m, 2H), 2.34–2.23 (m, 2H), 2.22–2.11 (m, 2H); ^13^C NMR (CDCl_3_): δ 166.6, 74.9, 23.2.

### Hydrolysis of Dilactone **3** to Obtain **2a**

Dilactone **3** (26 mg, 0.18 mmol) was dissolved in 2 mL of water and heated at 40 °C for 24 h. After this time, the solvent was removed, and **2a** was obtained as a white solid. *M*_p_ 135–136 °C. ^1^H NMR (D_2_O, 4.78 ppm): δ 4.40–4.34 (m, 2H), 2.05–1.94 (m, 2H), 1.89–1.76 (m, 2H). ^13^C NMR (D_2_O, spectrum of **2** used as reference): δ 178.2, 70.2, 29.5.

### Hydrogenation of **1** in THF and Hydrolysis

In a round-bottomed flask, 0.525 g of 3-hydroxy-2-pyrone-6-carboxylic acid (**1**) (purity 96%, 3.22 mmol) was dispersed in 7 mL of THF. Then, 0.250 g of 5% Pd/C (50% moisture) was added to this suspension, and the resulting mixture was stirred at room temperature under the hydrogen atmosphere (ambient pressure) until no further hydrogen consumption was observed (44 h). The reaction mixture was then filtered, and the solvent was removed under reduced pressure. A sample of the residue was analyzed by ^1^H NMR spectroscopy in the presence of terephthalic acid as an internal standard. To the crude (0.598 g), 21 mL of water was then added, and the mixture was heated at 90 °C for 4 h. After this time, solvent was removed under reduced pressure obtaining 0.620 g (purity 90%, 3.1 mmol), 96% yield of **2** (**2a**/**2b** = 95/5).

### Hydrogenation of **1** in Acetic Acid and Hydrolysis

In a round-bottomed flask, 0.516 g of 3-hydroxy-2-pyrone-6-carboxylic acid (**1**) (purity 96%, 3.16 mmol) was dispersed in 10 mL of acetic acid. Then, 0.253 g of 5% Pd/C (50% moisture) was added to this suspension and the resulting mixture was stirred at room temperature under the hydrogen atmosphere (ambient pressure) until no further hydrogen consumption was observed (25 h). The reaction mixture was then filtered, and the catalyst was washed 3 times with water. The aqueous solution recovered from the catalyst washing was combined with the acetic acid solution, and the solvents were removed under reduced pressure. A sample of the residue was analyzed by ^1^H NMR spectroscopy in the presence of terephthalic acid as an internal standard. To the crude, 20 mL of water was then added and the mixture was heated at 90 °C for 4 h. After this time, the solvent was removed obtaining 0.625 g (85% purity, 3.00 mmol), 95% yield of **2** (**2a**/**2b** = 96/4).

### Hydrogenation of **1** in Acetic Acid

In a round-bottomed flask, 0.500 g of 5% Pd/C (50% moisture) was dispersed and stirred in 11 mL of a solution 10:1 of acetic acid and acetic anhydride for 1 h. After this time, the suspension was decanted, and the liquid was removed. In a three-necked round-bottomed flask, 1.01 g of 3-hydroxy-2-pyrone-6-carboxylic acid (**1**) (purity 98%, 6.33 mmol) was dispersed in 15 mL of acetic acid. The catalyst was then dispersed in 5 mL of acetic acid, and the resulting suspension was added to that of pyrone **1**. Hydrogenation was carried out under stirring at room temperature (ambient pressure) until no further hydrogen consumption was observed. After 45 h, the mixture was filtered, two samples were withdrawn, and the solvent was removed, respectively, at 70 °C under reduced pressure and at room temperature under a stream of nitrogen. The samples were analyzed by ^1^H NMR spectroscopy.

### Hydrogenation of **1** in Ethanol

In a round-bottomed flask, 0.281 g of 5% Pd/C (50% moisture) was washed, under stirring, with 5 mL of di EtOH, decanted, and then dispersed in 8 mL of ethanol. The resulting suspension was transferred in a round-bottomed flask containing 0.557 g of 3-hydroxy-2-pyrone-6-carboxylic acid (**1**) (purity 96%, 3.42 mmol). The resulting mixture was stirred at room temperature under the hydrogen atmosphere (ambient pressure) until no further hydrogen consumption was observed for 9 h. The reaction mixture was then filtered, and the solvent was removed under reduced pressure, obtaining 0.824 g of an oil. A sample of the residue was analyzed by ^1^H NMR spectroscopy in the presence of terephthalic acid as an internal standard. Part of this crude (0.103 g) was purified by flash chromatography on silica gel (AcOEt/AcOH 95:5 as eluent) obtaining 0.016 g of 6-ethoxy-2,5-dihydroxy-6-oxohexanoic acid **7** (0.078 mmol) as an oil.^1^H NMR (DMSO-*d*_6_): δ 4.09 (dq, 2H, *J* = 7.1 and 1.0 Hz), 4.03–3.98 (m, 1H), 3.95–3.91 (m, 1H), 1.77–1.66 (m, 2H), 1.63–1.52 (m, 2H), 1.19 (t, 3H, *J* = 7.1 Hz); ^13^C NMR (DMSO-*d*_6_): δ 175.6, 174.0, 69.4, 69.1, 59.9, 29.6, 29.5, 14.1; MS/MS (ESI): *m*/*z* 204.9 (15) [M – H]®, 158.9 (100) [M – EtOH-H]®.

### Hydrogenation of **1** in Ethanol and Hydrolysis

In a round-bottomed flask, 2.06 g of 3-hydroxy-pyran-2-one-6-carboxylic acid (**1**) (purity 96%, 12.6 mmol) was dispersed in 40 mL of ethanol. Then, 1.01 g of 5% Pd/C (50% moisture) was added to this suspension, and the resulting mixture was stirred at room temperature under the hydrogen atmosphere (ambient pressure) for 24 h. The reaction mixture was then filtered, and the solvent was removed under reduced pressure. To the crude, 30 mL of water and 1 mL of trifluoroacetic acid were added and the mixture was heated at 90 °C for 5 h. After this time, the solvent was removed obtaining 2.76 g (74% purity, 11.4 mmol, presence of residual water as impurity), 90% yield of **2** (**2a**/**2b** = 96/4).

### Hydrogenation of **1** in THF in H-Cube Apparatus

In a round-bottomed flask, 62.5 mg of **1** (purity 98% 0.393 mmol) was dissolved under stirring in 70 mL of THF and transferred in the H-Cube apparatus. The reaction was performed at 30 °C and at a pressure of 10 bar with a flow of the THF solution of 1 mL/min through a cartridge containing 1.2 cm^3^ of 10% Pd/C. Samples were withdrawn at the times as reported in Table S2, the solvent was removed and the residues were analyzed by ^1^H NMR spectroscopy using terephthalic acid as an internal standard. Results are reported in Table S2. ^1^H NMR (DMSO-*d*_6_) of **6a**: δ 4.97 (m, 1H), 4.22 (dd, 1H, *J* = 11.0 and 7.3), 2.25–2.05 (m, 2H), 2.00–1.90 (m, 1H), 1.65–1.52 (m, 1H).

### Synthesis of Dilactone **3** by Thermal Dehydration of a 95/5 Mixture of **2a**/**2b** in Tinyclave

In a 25 mL tinyclave, a 93/7 mixture of **2a**/**2b** (0.500 g, purity 77%, 2.15 mmol, obtained from the hydrolysis of the reaction mixture prepared by hydrogenation in THF) was dissolved in 20 mL of acetic acid. The reaction was heated in an oil bath set at 140 °C under stirring for 5 h. After this time, the mixture was cooled to room temperature and the solvent was removed under reduced pressure to obtain a white solid. The solid was transferred in a sublimation apparatus, heated in an oil bath at 140 °C at a pressure of 0.3 Torr recovering **3** as a white solid (0.173 g, purity 86%, 1.22 mmol, yield 60%.

### Reaction of Dilactone **3** and Diamine **8**

In a round-bottomed flask, 11.7 mg of dilactone **3** (purity 85%, 0.0700 mmol) and 9.23 mg of 1,6-hexamethylenediamine (**8**) (0.0794 mmol) were dissolved in 0.4 mL of DMSO-*d*_6_ and the resulting mixture was stirred at room temperature for 24 h. After this, ^1^H NMR (Figure 6) and ^13^C NMR (Figure S15) spectra were recorded. ^1^H NMR (DMSO-*d*_6_): δ 7.78–7.55 (m, 2H); 3.88–3.75 (m, 2H), 3.23–2.84 (m, 4H), 2.71 (t, 0.3, two end groups C*H*_2_NH_2_), 1.75–1.58 (m, 2H), 1.57–1.44 (m, 2H), 1.44–1.31 (m, 4H), 1.28–1.14 (m, 4H); ^13^C NMR (DMSO-*d*_6_): δ 174.7, 71.8, 39.0, 31.3, 30.1, 27.0 ppm. To the DMSO-*d*_6_ solution, 0.4 mL of chloroform was added, obtaining a white solid. This latter was filtered, washed with chloroform, and ATR-FTIR was recorded (Figure S17). ATR-FTIR: ν 3387, 3295, 2924, 2855, 1630, and 1534 cm^–1^.

## Supplementary Material



## Notes

Representative ^1^H NMR and ^13^C NMR spectra, MS spectrum of the oligomer, and schemes (PDF)

## Abbreviations

DHAA: dihydroxyadipic acid
AA: adipic acid
THFDCA: tetrahydrofuran-2,5-dicarboxylic acid
