# Supplementary material for: Facile Diastereoselective Synthesis of Dihydroxyadipic Acid and Dihydroxyadipic Dilactone by Catalytic Reduction of Biosourced 3‑Hydroxy-2-Pyrone-6-Carboxylic Acid
Source: ACS Omega. 2025 Jul 7;10(28):30660–9. doi: 10.1021/acsomega.5c02695 (PMC12290715; doi:10.1021/acsomega.5c02695)
Supplement: Supplementary file 1 [file ao5c02695_si_001.pdf]

Supporting Information

for

Facile diastereoselective synthesis of dihydroxyadipic acid and dihydroxyadipic dilactone by catalytic reduction of biosourced 3-hydroxy-2-pyrone-6-carboxylic acid

Gabriella Leonardi, Aurora Bertuzzi, Ada Truscello, Cristian Gambarotti, Roberto Sebastiano*

Department of Chemistry, Materials and Chemical Engineering "Giulio Natta", Politecnico di Milano, piazza Leonardo da Vinci 32, I-20133 Milano, Italy.

Number of pages: 14

Number of tables: 1

Number of figures: 17

Number of schemes: 4

List of tables

- **Table S1**: Hydrogenation of pyrone **1** in the H-Cube apparatus

NMR spectra of isolated products

- **Figure S1**: ^{1}H NMR (D_2O) spectrum of DHAA **2**
- **Figure S2**: ^{13}C NMR (D_2O) spectrum of DHAA **2**
- **Figure S3**: ^{1}H NMR ($CDCl_3$) spectrum of dilactone **3**
- **Figure S4**: ^{13}C NMR ($CDCl_3$) spectrum of dilactone **3**
- **Figure S5**: ^{1}H NMR (D_2O) spectrum of DHAA **2a**
- **Figure S6**: ^{13}C NMR (D_2O) spectrum of DHAA **2a**
- **Figure S7**: ^{1}H NMR (DMSO-d6) spectrum of monoester **7**
- **Figure S8**: ^{13}C NMR (DMSO-d6) spectrum of monoester **7**

NMR spectra of reaction mixtures

- **Figure S9**: ^{1}H NMR (DMSO-d6) spectrum of the reaction mixture obtained from hydrogenation of **1** in THF
- **Figure S10**: ^{1}H NMR (DMSO-d6) spectrum of the reaction mixture obtained from hydrogenation of **1** in acetic acid
- **Figure S11**: ^{1}H NMR (DMSO-d6) spectra of the reaction mixture obtained from hydrogenation of 1 in acetic acid: a) evaporation by rotavapor at 70 °C (red) compared with evaporation of acetic acid by nitrogen flux (blue)
- **Figure S12**: ^{1}H NMR (DMSO-d6) spectrum of the reaction mixture obtained from hydrogenation of **1** in ethanol
- **Figure S13**: ^{13}C NMR (D_2O) spectra of the reaction mixtures reported in table 1: a) reaction in water; b) reaction in THF and hydrolysis; c) reaction in acetic acid and hydrolysis; d) reaction in ethanol and hydrolysis
- **Figure S14**: ^{1}H NMR (DMSO-d6) spectra of the samples withdrawn at different times from reaction in H Cube
- **Figure S15:** ^{13}C NMR (DMSO-d_6) spectrum of the polymerization mixture

ESI-MS spectrum

- **Figure S16**: ESI-MS spectrum of the polymerization mixture

ATR-FTIR spectrum

Figure S17: ATR-FTIR spectrum of the solid precipitated from the reaction mixture

Schemes

- **Scheme S1**. Possible evolution of the mono reduced lactones **4a’’** to dihydroxyadipic acid **2**
- **Scheme S2**. Possible evolution of **4b’** to dihydroxyadipic acid **2**
- **Scheme S3.** Catalytic hydrogenation of **1** (through formation of **4a’’** and **6a’’**): general scheme
- **Scheme S4.** Catalytic hydrogenation of **1** (through formation of **4b’** and **6a’**): general scheme

Table S1 – Hydrogenation of pyrone **1** in the H-Cube apparatus[a]: yields[b] of products.

Entry	Time (min)	Pyrone **1** (%)	Yield of **6a** (%)	Yield of **2** (%)	Yield of **3** (%)
1	0	100	0	0	0
2	60	71	24	1	n.d.[c]
3	120	50	42	n.d.[c]	n.d.[c]
4	180	32	63	n.d.[c]	1
5	240	17	77	n.d.[c]	1
6	345	2	85	2	1

a) Hydrogenation performed in THF, at 10 bar, 30 °C in the presence of 10% Pd/C; b) analytical yields were calculated by ^{1}H NMR analysis in the presence of an internal standard; c) not detected by ^{1}H NMR analysis.

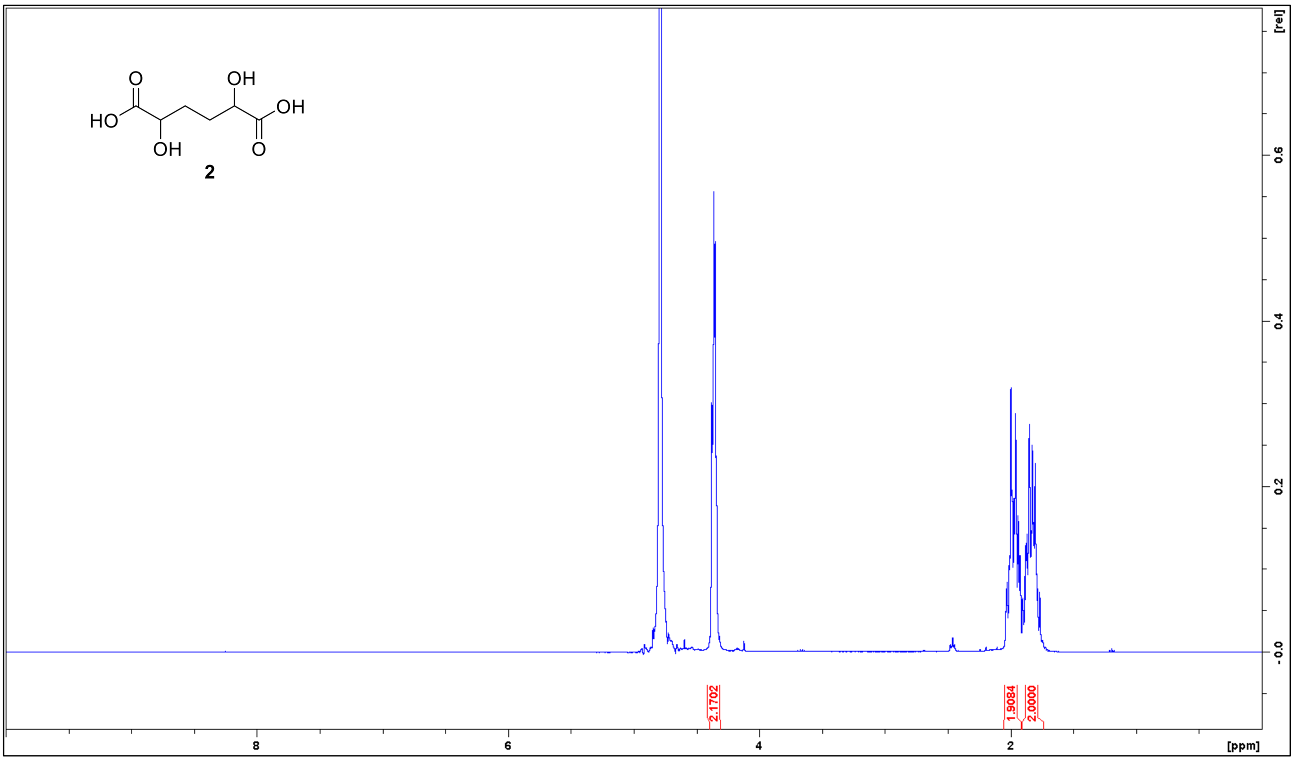

Figure S1: ^{1}H NMR (D_2O) spectrum of DHAA **2**

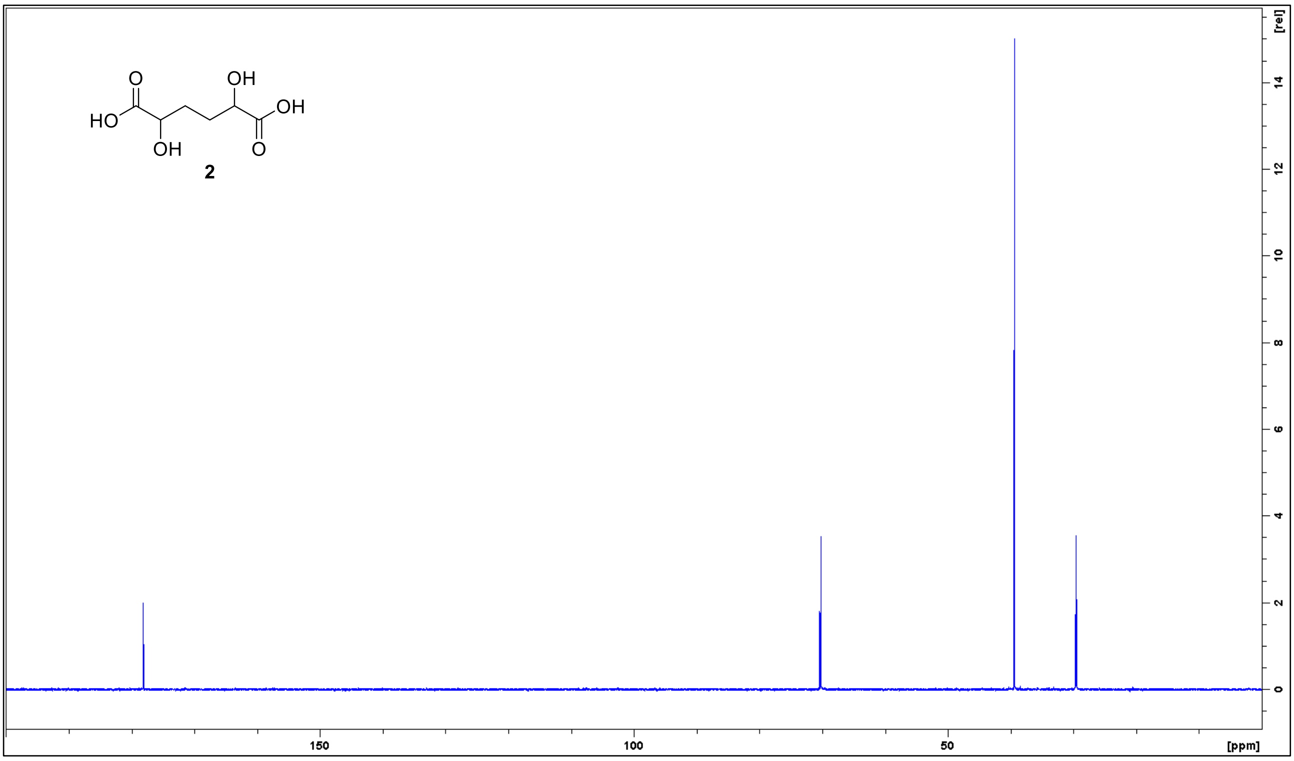

Figure S2: ^{13}C NMR (D_2O + DMSO as reference 39.39 ppm) spectrum of DHAA **2**

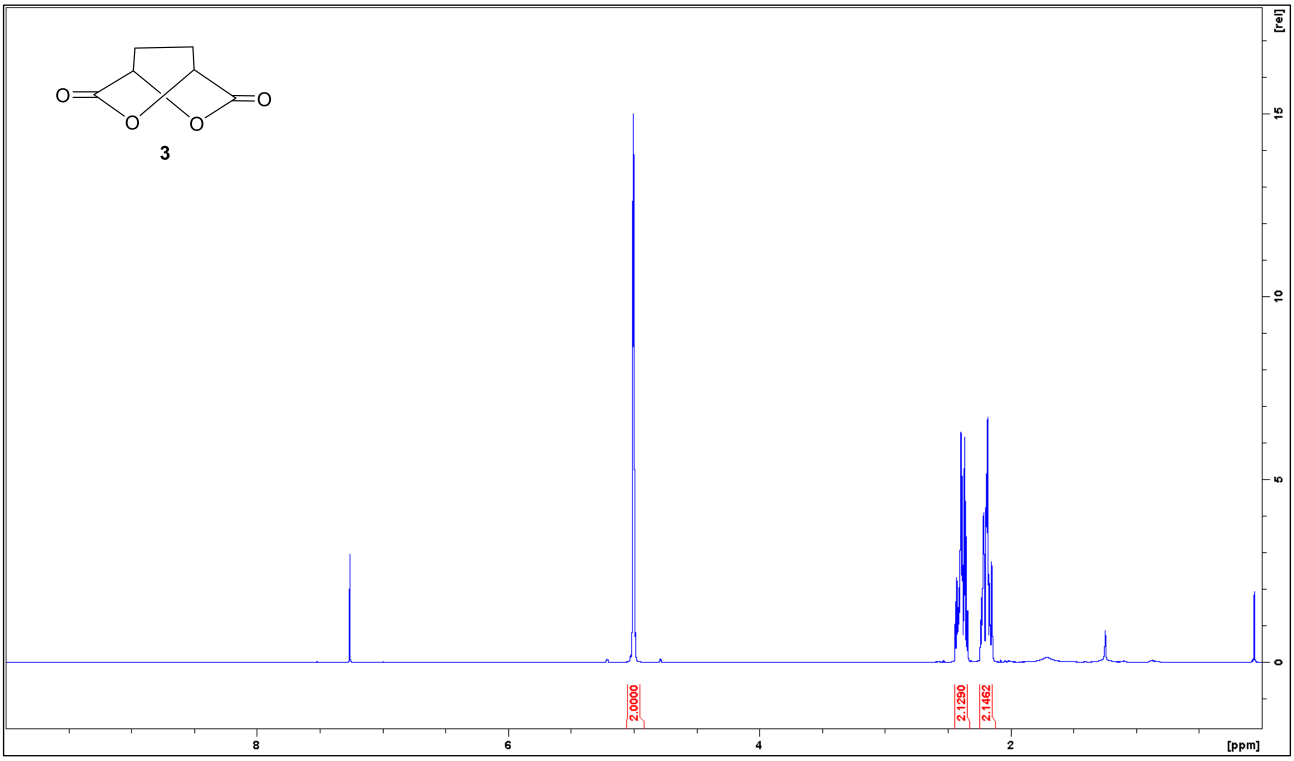

Figure S3: ^{1}H NMR ($CDCl_3$) spectrum of dilactone **3**

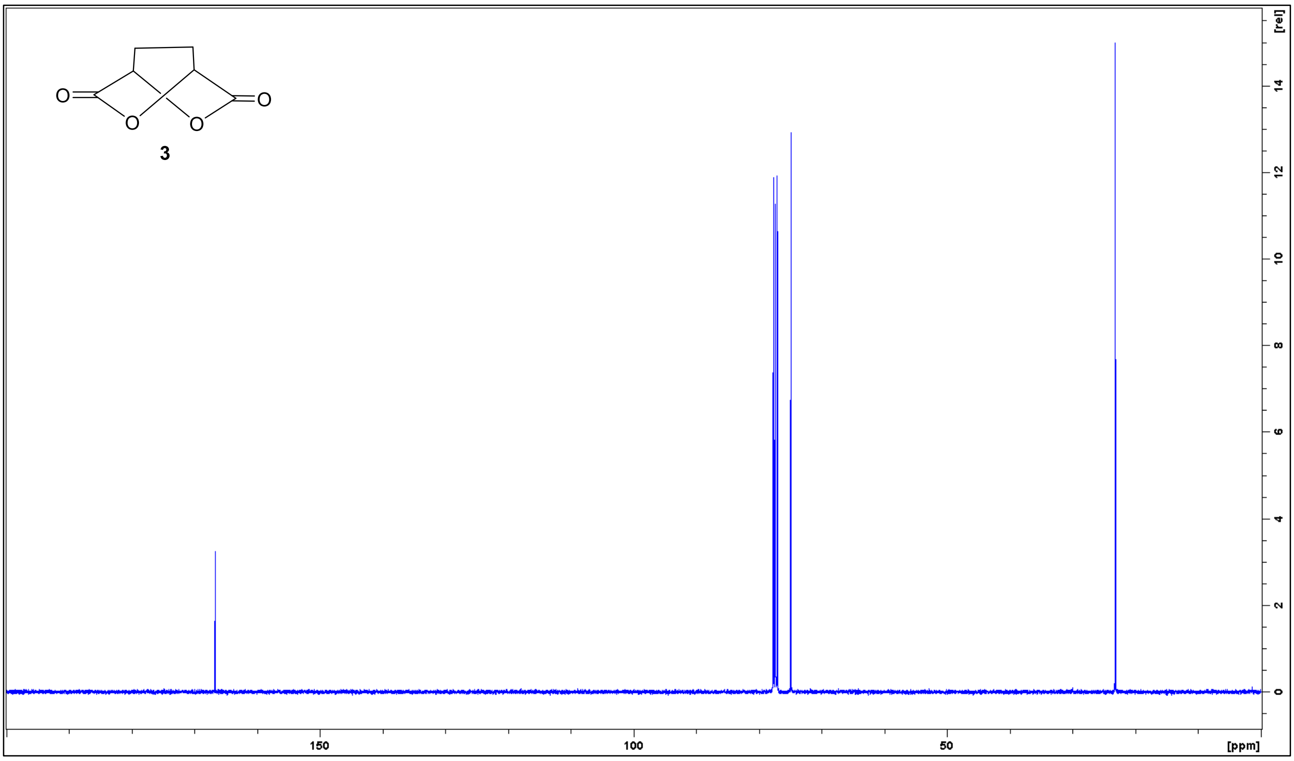

Figure S4: ^{13}C NMR ($CDCl_3$) spectrum of dilactone **3**

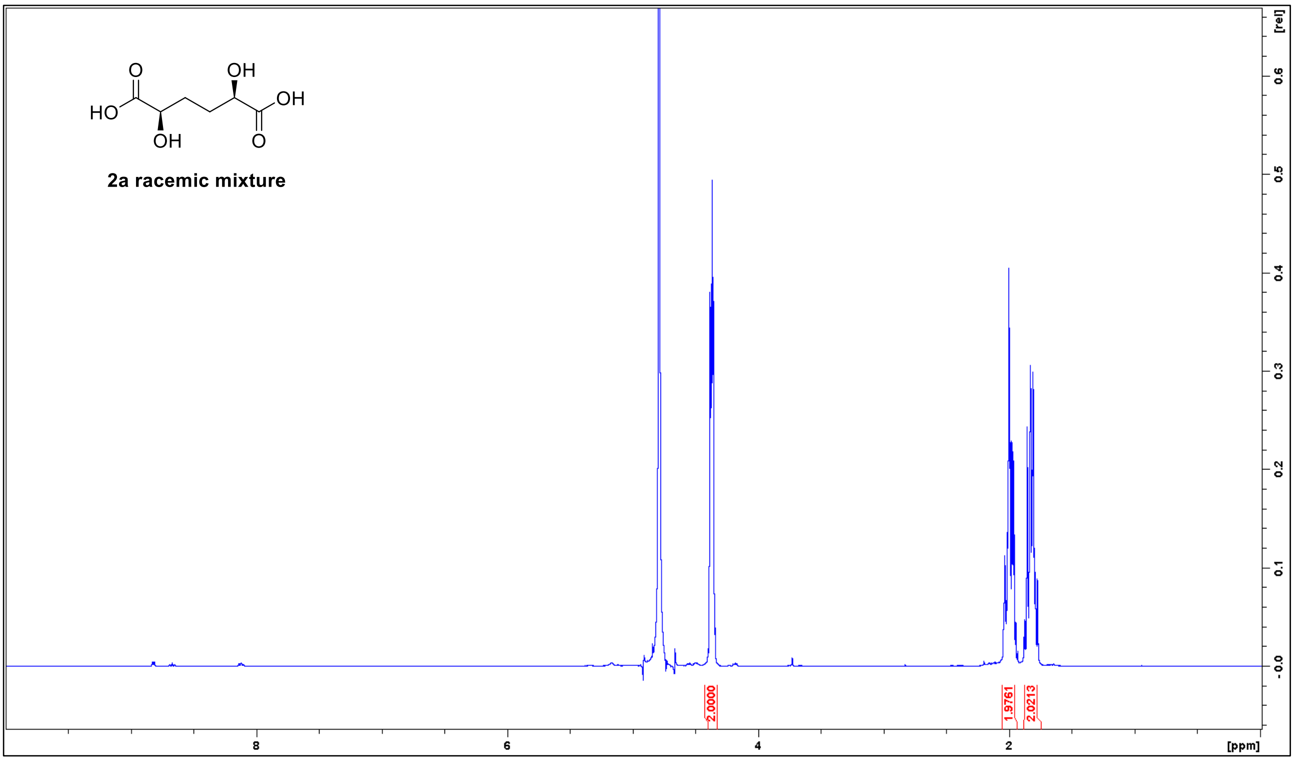

Figure S5: ^{1}H NMR (D_2O) spectrum of DHAA **2a**

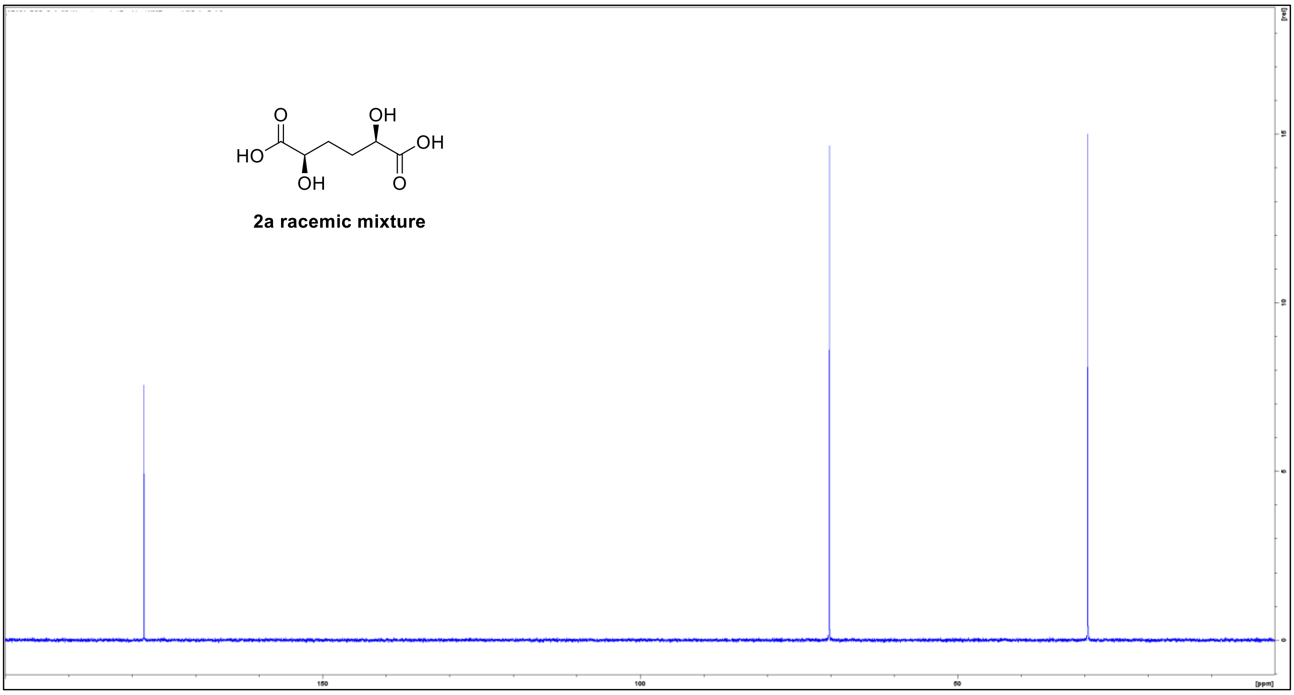

Figure S6: ^{13}C NMR (D_2O) spectrum of DHAA **2a**

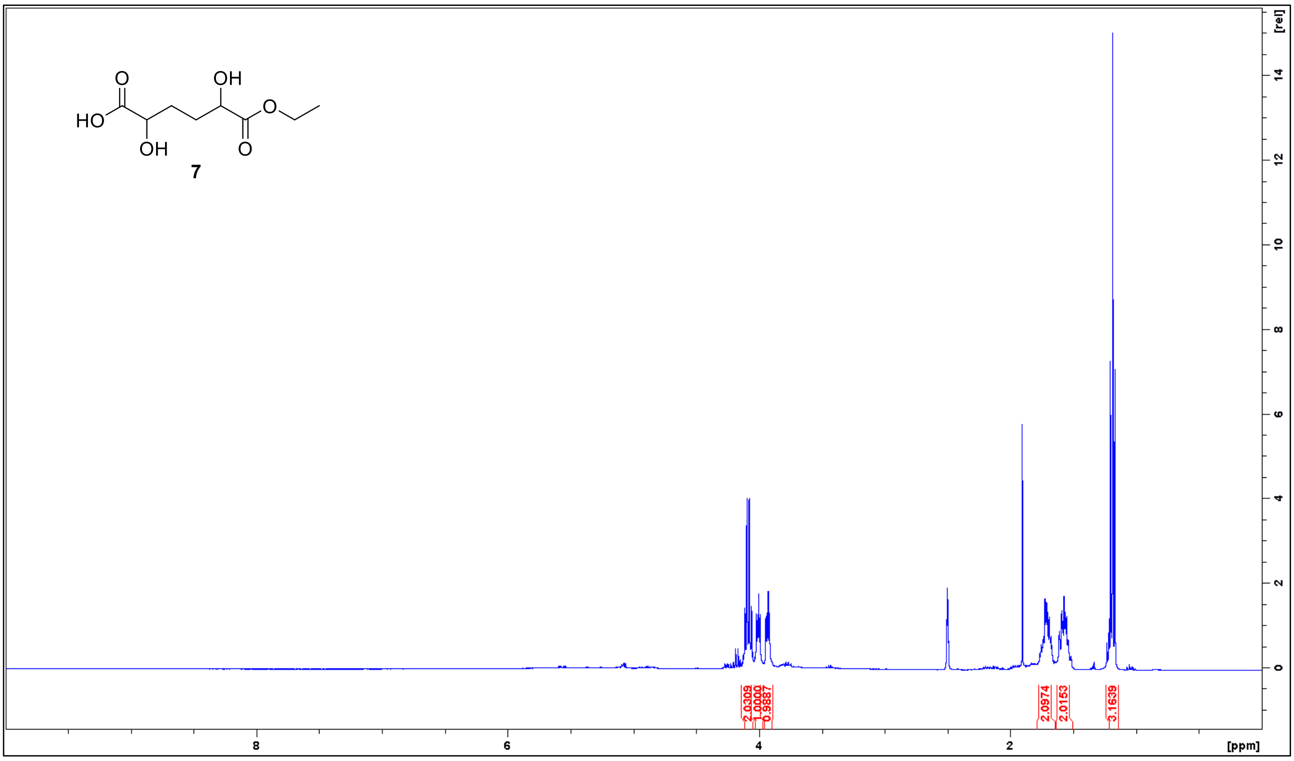

Figure S7: ^{1}H NMR (DMSO-d6) spectrum of monoester **7**

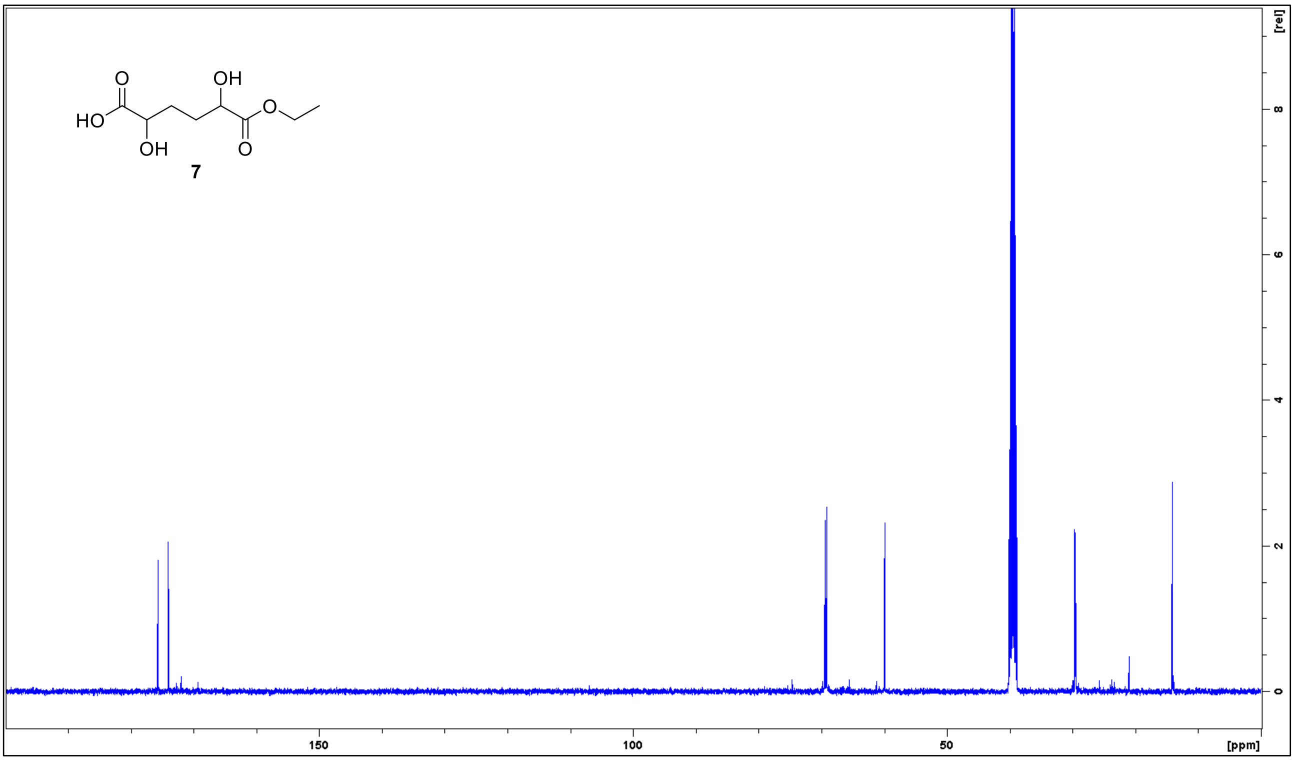

Figure S8: ^{13}C NMR (DMSO-d6) spectrum of monoester **7**

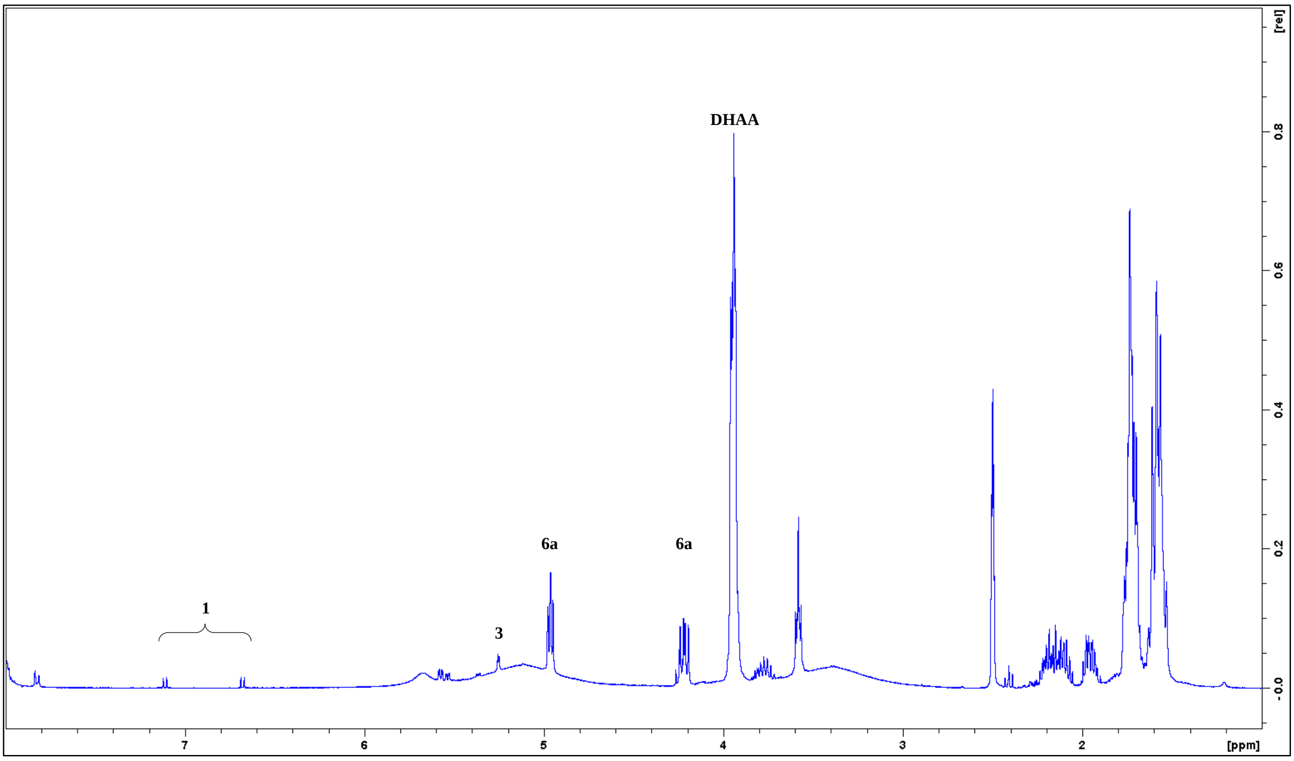

Figure S9: ^{1}H NMR (DMSO-d_6) spectrum of the reaction mixture obtained from hydrogenation of **1** in THF

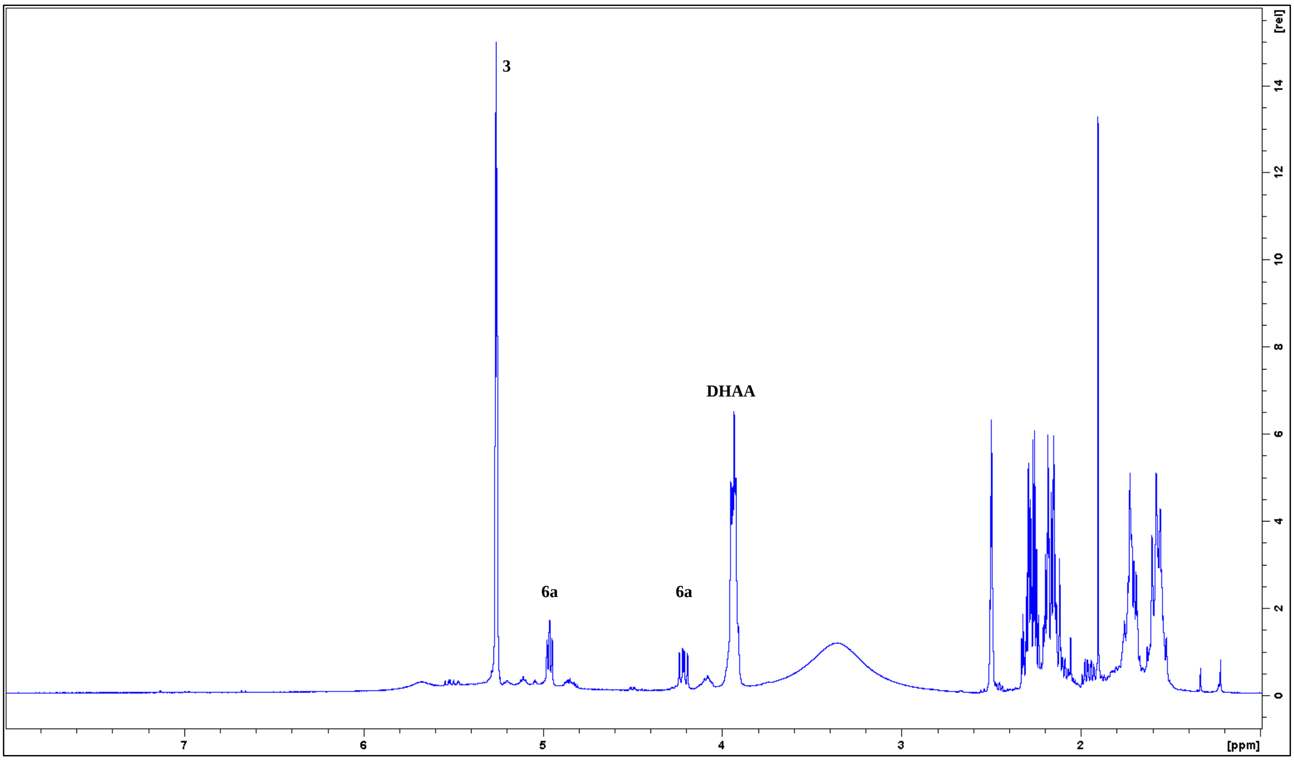

Figure S10: ^{1}H NMR (DMSO-d$_6$) spectrum of the reaction mixture obtained from hydrogenation of **1** in acetic acid

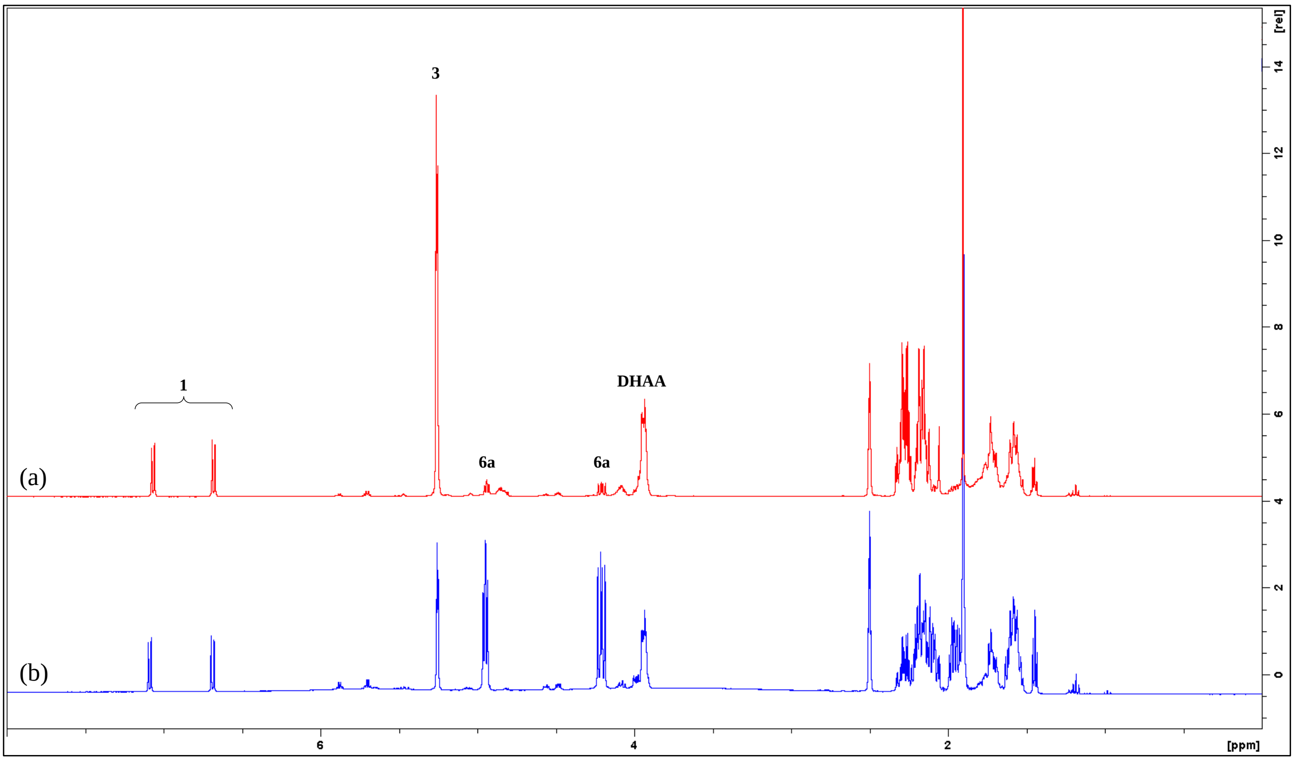

Figure S11: ^{1}H NMR (DMSO-d$_6$) spectra of the reaction mixture obtained from hydrogenation of **1** in acetic acid: a) evaporation by rotavapor at 70 °C (red) compared with evaporation of acetic acid by N_2 flux (blue)

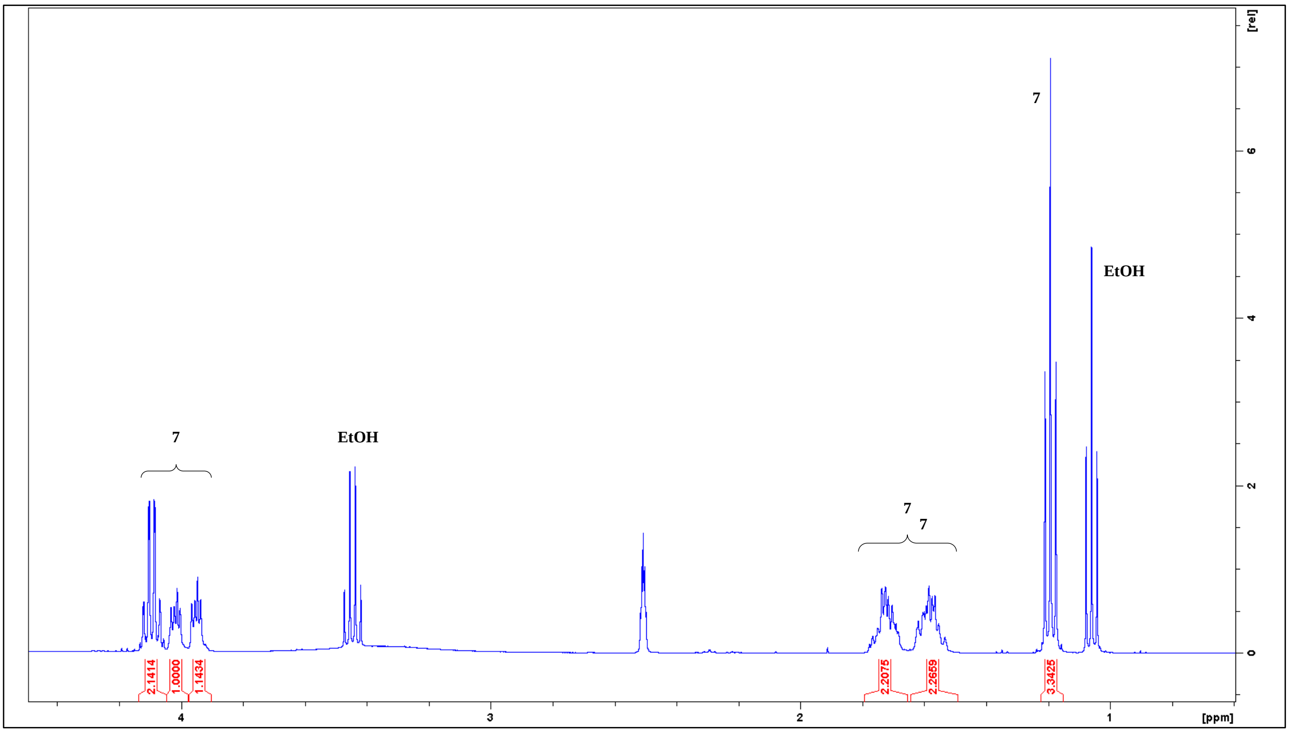

Figure S12: ^{1}H NMR (DMSO-d_6) spectrum of the reaction mixture obtained from hydrogenation of **1** in ethanol

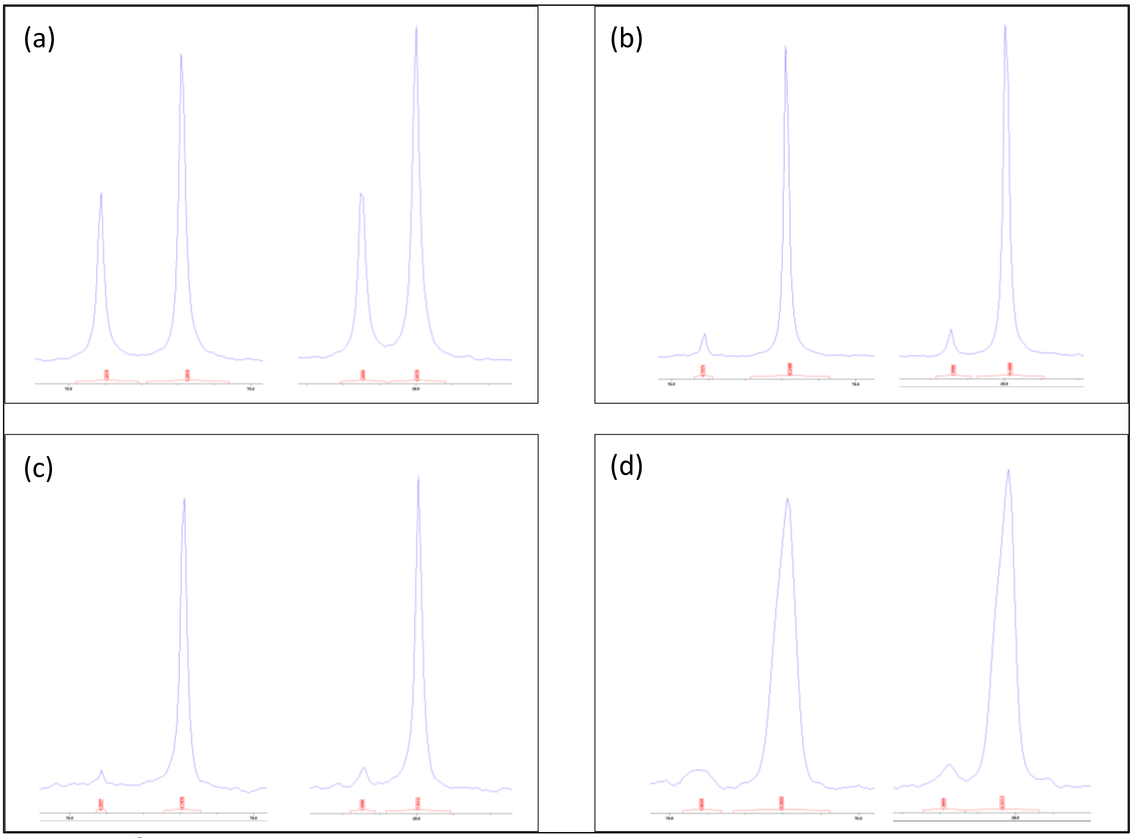

Figure S13: ^{13}C NMR (D_2O) spectra of the reaction mixtures reported in table 1: a) reaction in water; b) reaction in THF and hydrolysis; c) reaction in acetic acid and hydrolysis; d) reaction in ethanol and hydrolysis

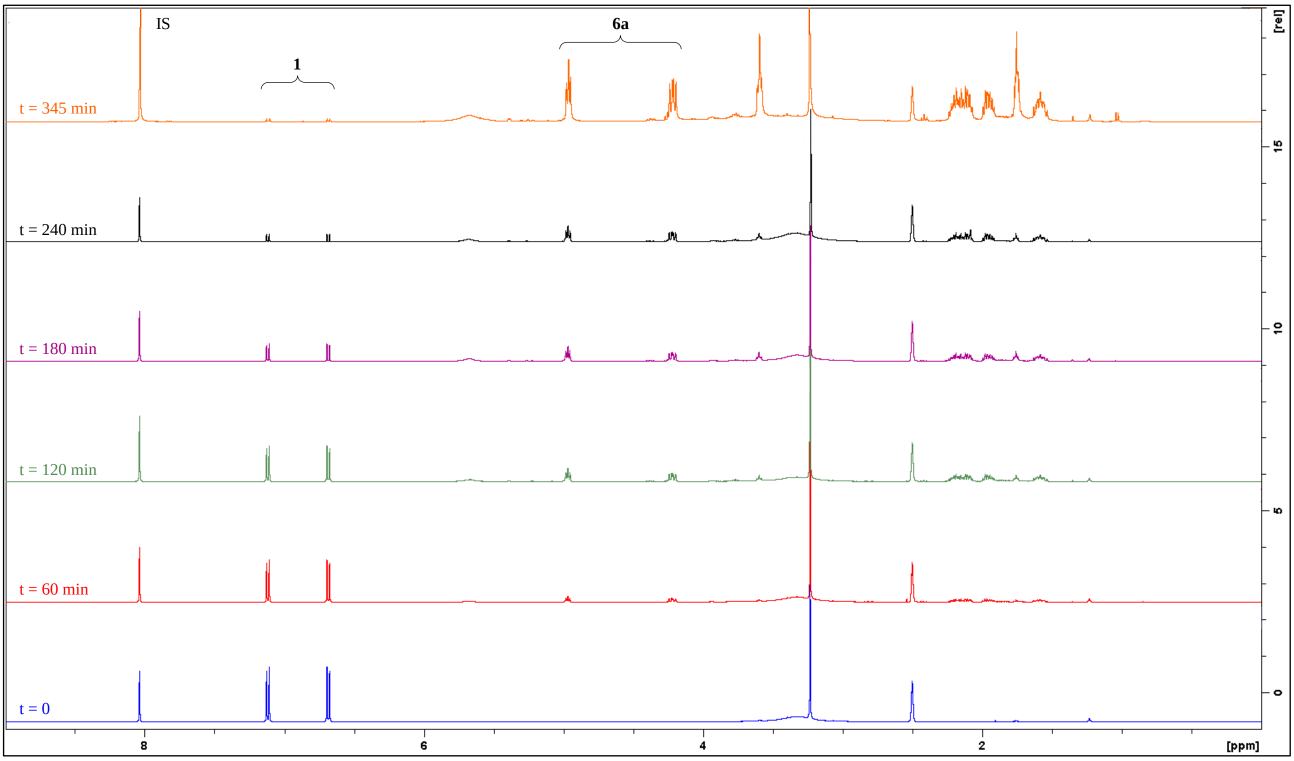

Figure S14: ^{1}H NMR (DMSO-d$_6$) spectra of the samples withdrawn at different times from reaction in H Cube

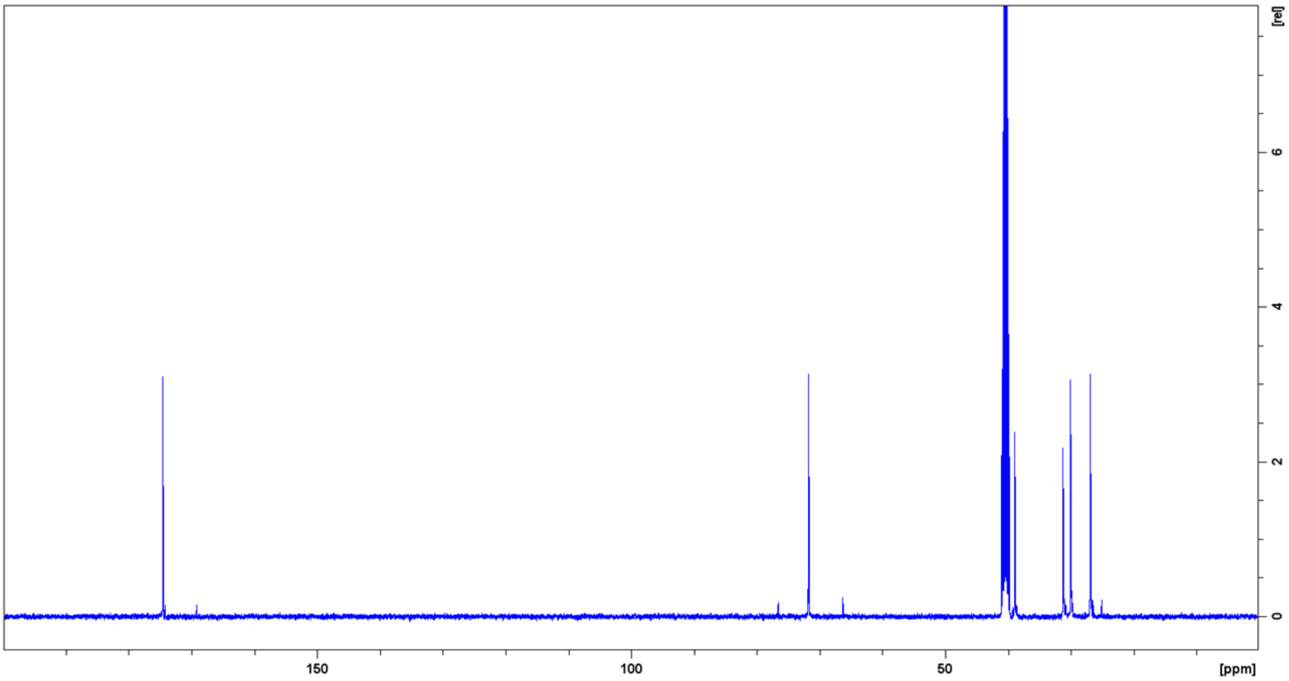

Figure S15: ^{13}C NMR (DMSO-d$_6$) spectrum of the polymerization mixture

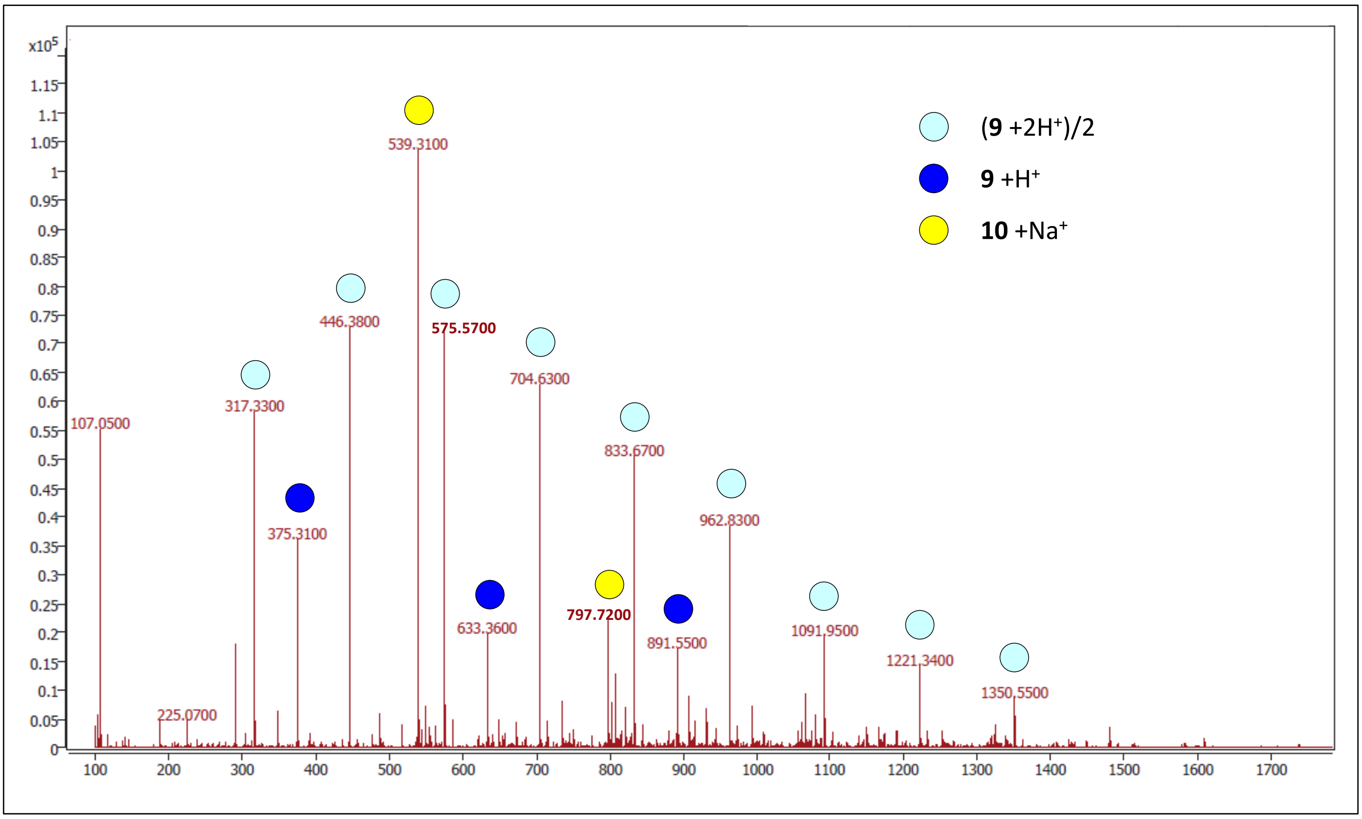

Figure S16: ESI-MS of the polymerization mixture

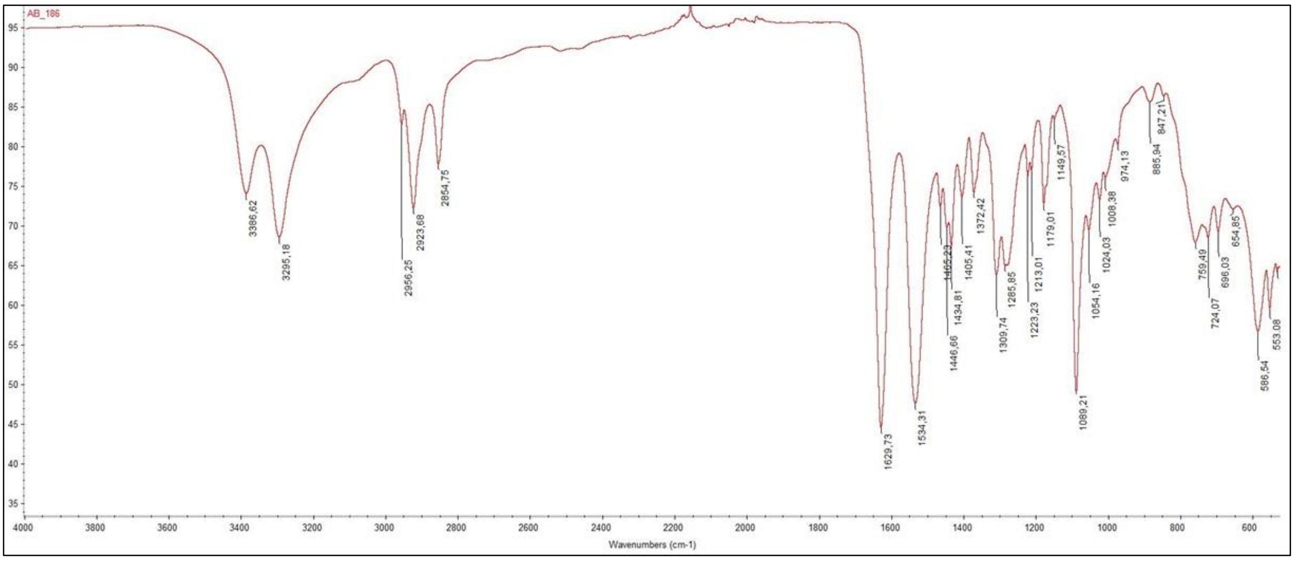

Figure S17: ATR-FTIR spectrum of the solid precipitated from the reaction mixture

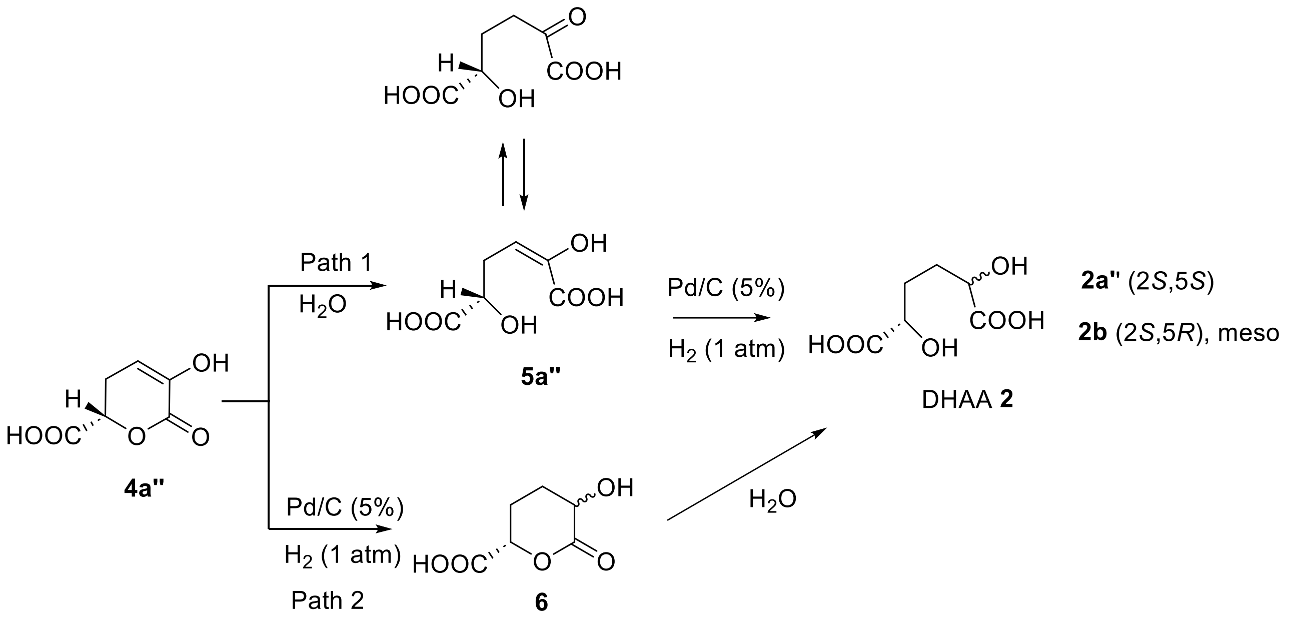

Scheme S1. Possible evolution of the mono reduced lactones **4a''** to dihydroxyadipic acid **2**

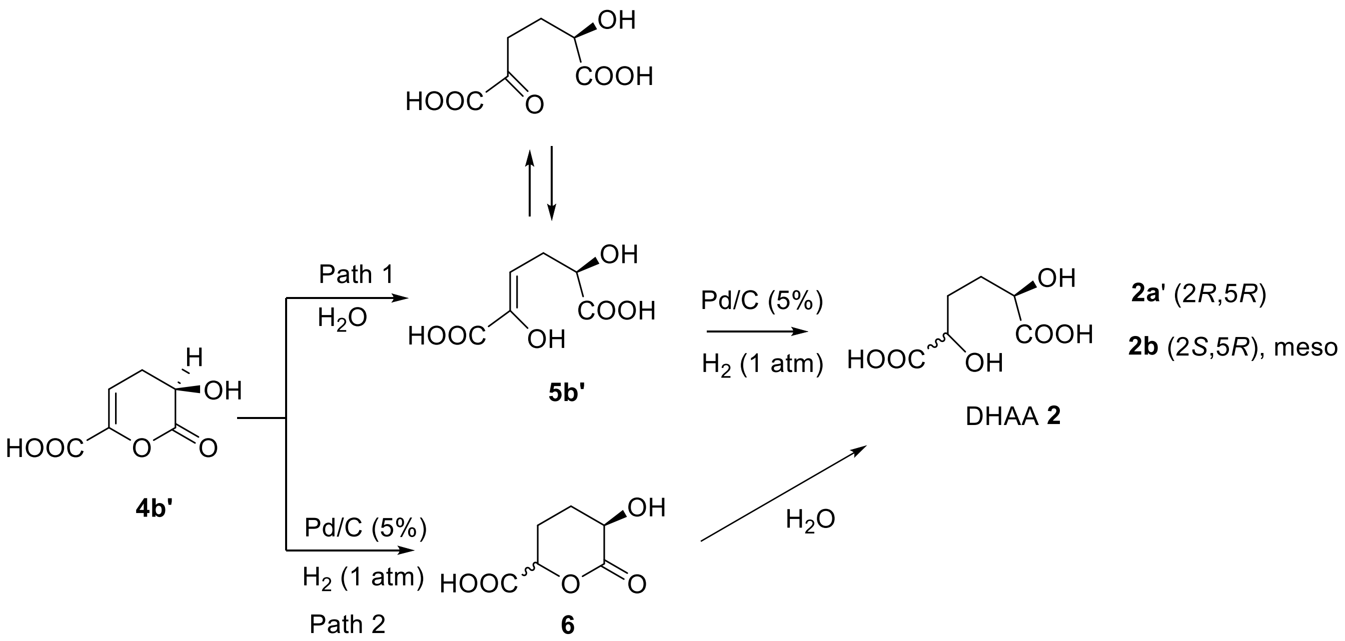

Scheme S2. Possible evolution of **4b'** to dihydroxyadipic acid **2**. Only the enantiomer *R* (**4b'**) is represented in the scheme; the enantiomer *S* (**4b''**) leads to the corresponding products

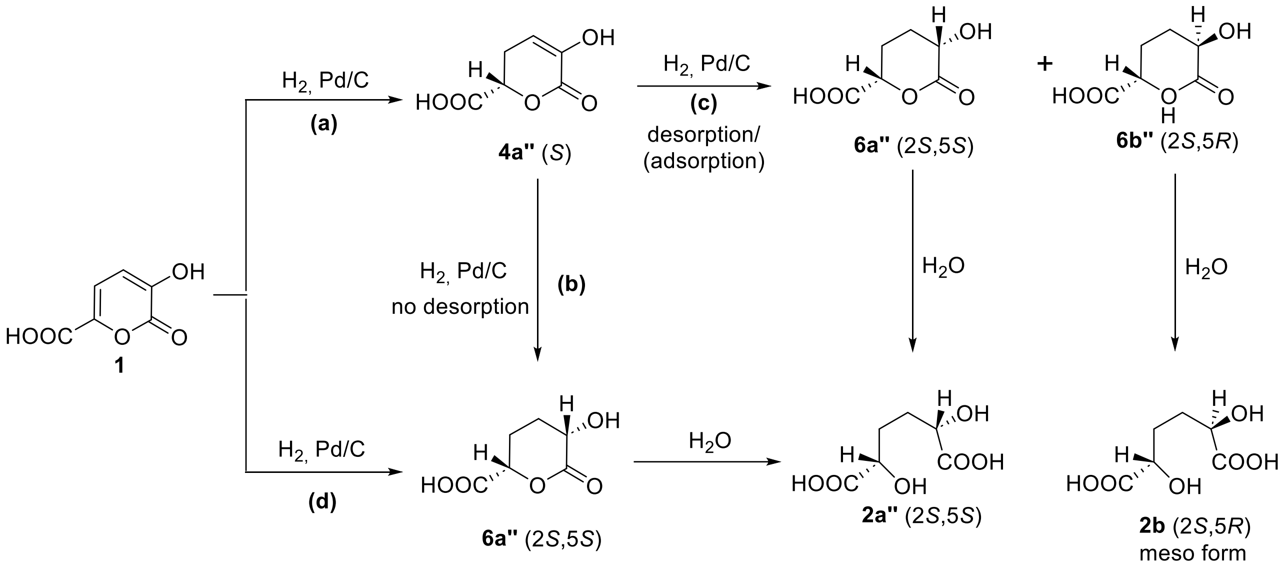

Scheme S3. Catalytic hydrogenation of **1** (through formation of **4a''** and **6a''**): general scheme

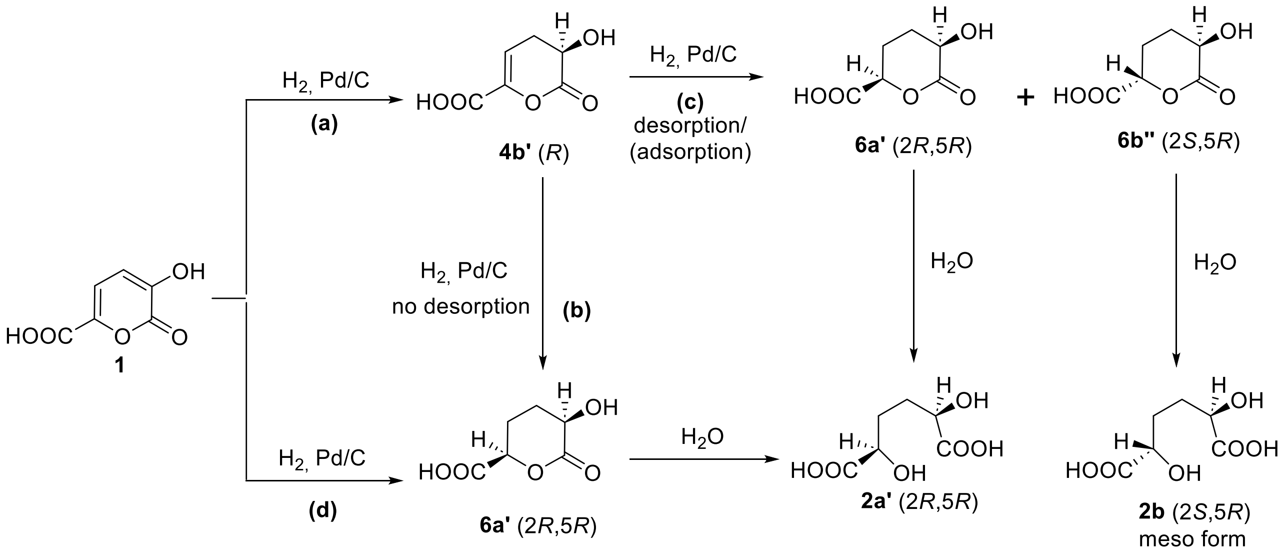

Scheme S4. Catalytic hydrogenation of **1** (through formation of **4b'** and **6a'**): general scheme. Only the enantiomer *R* (**4b'**) is represented in the scheme; the enantiomer *S* (**4b''**) leads to the corresponding products
